# A Genetic Bottleneck of Mitochondrial DNA During Human Lymphocyte Development

**DOI:** 10.1093/molbev/msac090

**Published:** 2022-04-28

**Authors:** Zhongjie Tang, Zhaolian Lu, Baizhen Chen, Weixing Zhang, Howard Y. Chang, Zheng Hu, Jin Xu

**Affiliations:** State Key Laboratory of Biocontrol, School of Life Sciences, Sun Yat-Sen University, Guangzhou, China; CAS Key Laboratory of Quantitative Engineering Biology, Shenzhen Institute of Synthetic Biology, Shenzhen Institute of Advanced Technology, Chinese Academy of Sciences, Shenzhen, China; State Key Laboratory of Biocontrol, School of Life Sciences, Sun Yat-Sen University, Guangzhou, China; State Key Laboratory of Biocontrol, School of Life Sciences, Sun Yat-Sen University, Guangzhou, China; Center for Personal Dynamic Regulomes, Stanford University, Stanford, CA, USA; Howard Hughes Medical Institute, Stanford University, Stanford, CA, USA; CAS Key Laboratory of Quantitative Engineering Biology, Shenzhen Institute of Synthetic Biology, Shenzhen Institute of Advanced Technology, Chinese Academy of Sciences, Shenzhen, China; State Key Laboratory of Biocontrol, School of Life Sciences, Sun Yat-Sen University, Guangzhou, China

**Keywords:** mitochondrial DNA, somatic genetic bottleneck, lymphocyte, scATAC-seq

## Abstract

Mitochondria are essential organelles in eukaryotic cells that provide critical support for energetic and metabolic homeostasis. Although the elimination of pathogenic mitochondrial DNA (mtDNA) mutations in somatic cells has been observed, the mechanisms to maintain proper functions despite their mtDNA mutation load are poorly understood. In this study, we analyzed somatic mtDNA mutations in more than 30,000 single human peripheral and bone marrow mononuclear cells. We observed a significant overrepresentation of homoplasmic mtDNA mutations in B, T, and natural killer (NK) lymphocytes. Intriguingly, their overall mutational burden was lower than that in hematopoietic progenitors and myeloid cells. This characteristic mtDNA mutational landscape indicates a genetic bottleneck during lymphoid development, as confirmed with single-cell datasets from multiple platforms and individuals. We further demonstrated that mtDNA replication lags behind cell proliferation in both pro-B and pre-B progenitor cells, thus likely causing the genetic bottleneck by diluting mtDNA copies per cell. Through computational simulations and approximate Bayesian computation (ABC), we recapitulated this lymphocyte-specific mutational landscape and estimated the minimal mtDNA copies as <30 in T, B, and NK lineages. Our integrative analysis revealed a novel process of a lymphoid-specific mtDNA genetic bottleneck, thus illuminating a potential mechanism used by highly metabolically active immune cells to limit their mtDNA mutation load.

## Introduction

Mitochondria are essential cellular organelles that orchestrated a wide range of biological processes, including energy production and oxidative metabolism (Chandel 2014). Immune cells have high and dynamic metabolic requirements for their immune functions in healthy and diseased states, which rely on the mitochondrial biogenesis. For instance, the mitochondrial dynamics controls T cell fate through metabolic programming (Buck et al. 2016). Extensive mitochondrial remodeling, including alterations in morphology and increases in the copy numbers of mitochondrial DNA (mtDNA) during T cell activation has been reported (Tan et al. 2017; Mimitou et al. 2021). The metabolic alterations in immune cells have been observed not only in severe acute respiratory syndrome coronavirus 2 (SARS-CoV-2) infection (Lee et al. 2022), but also in the antitumor activity of tumor-infiltrating T lymphocytes (Yu et al. 2020). The mitochondrial translation is required for sustained killing by cytotoxic T cells and tumor-infiltrating T lymphocytes with depolarized mitochondria display characteristics of terminally exhaustion (Yu et al. 2020; Lisci et al. 2021).

The mitochondria function relies on the mtDNA, which encodes 13 core peptide subunits of the oxidative phosphorylation system and 24 RNAs involved in intramitochondrial protein synthesis (Andersion et al. 1981, Andrews et al. 1999). Typically, hundreds to thousands of copies of mtDNA molecules are present in each cell, and the germline mtDNA is predominantly maternally inherited and does not undergo recombination (Hagström et al. 2014). MtDNA accumulates mutations at a rate that is 5–10 times higher per site than that in the nuclear genome, because of the lack of DNA repair systems and frequent contact with mutagenic reactive oxygen species (Kazak et al. 2012; Lagouge and Larsson 2013; Scheibye-Knudsen et al. 2015). More than 500 pathogenic mtDNA mutations have been identified as genetic defects causing various human diseases (Ye et al. 2014). According to the theory known as “Muller’s ratchet,” continual accumulation of deleterious mutations in the absence of purifying selection leads to a decline in population fitness and will ultimately result in mutational meltdown (Felsenstein 1974). To avoid this outcome, the animal germline has evolved a mitochondrial genetic bottleneck, wherein only a small subset of mtDNA is transmitted to the next generation, thus resulting in substantial removal of deleterious mutations (Koehler et al. 1991; Ghosh et al. 1996; Jenuth et al. 1996).

A recent case report had shown that the pathogenic mutation 3243A/G, the cause of mitochondrial myopathy, encephalopathy, lactic acidosis, and stroke-like episode (Walker et al. 2020), was remarkably purified in lymphoid immune cells, particularly in T cells, as compared with other blood cells from peripheral blood mononuclear cells (PBMCs). A rare blood sample from a patient with Pearson’s syndrome in an acute anemic state caused by mtDNA mutations, spontaneously recovered with clearance of erythroid mitochondrial (Ahlqvist et al. 2015). These observations rose the questions on how pathogenic mtDNA mutations are inherited in somatic cells and how they affect the immune system.

To answer these questions, understanding of the clonal dynamics of mtDNA in the development of somatic cell lineages is necessary; however, systematical and quantitative studies are lacking due to the technical limitations of detecting heteroplasmic mutations in single cells. We and others have recently developed a single-cell lineage tracing method leveraging the somatic mtDNA mutations detected in single-cell assay for transposase-accessible chromatin with high-throughput sequencing (scATAC-seq) and/or RNA-seq (scRNA-seq) data (Ludwig et al. 2019; Xu et al. 2019). Using this method, we systematically investigated the mtDNA mutation landscape in 30,000 human single peripheral and bone marrow mononuclear cells (BMMCs). We revealed a novel process of genetic bottleneck in lymphoid lineage development and quantified the size of the genetic bottleneck with the approximate Bayesian computation (ABC) method. Together, our results demonstrated a novel mechanism that may strengthen purifying selection and consequently enable better quality control of their mitochondrial genomes, owing to the metabolic needs for immune responses.

## Results

### Somatic Mutational Landscape of mtDNA at Single-Cell Resolution

**Fig. 1.**
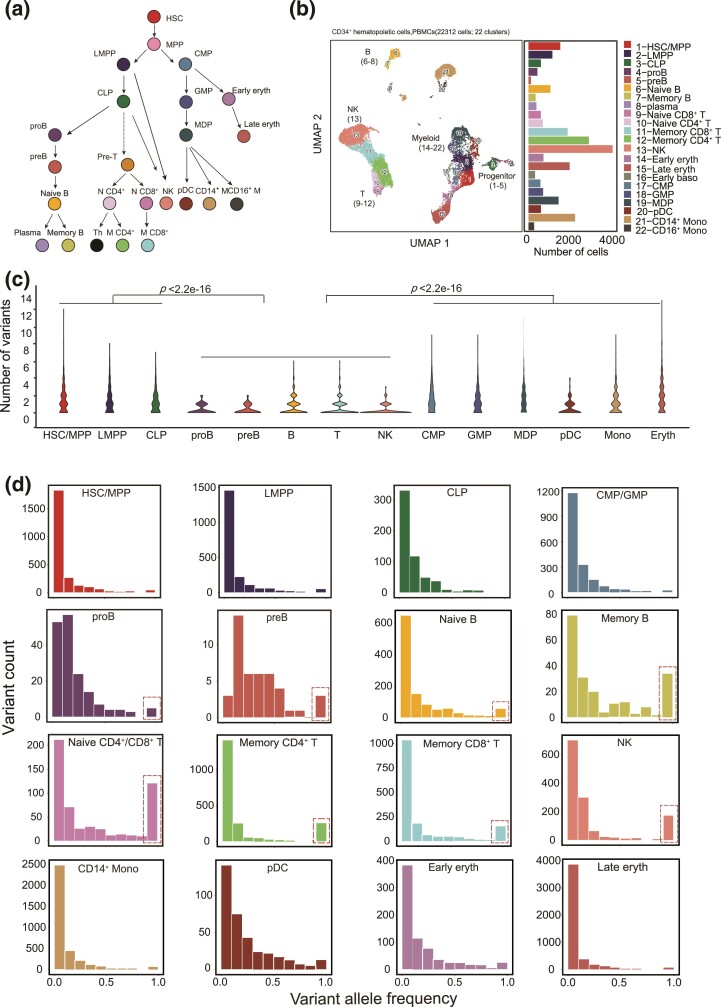
Somatic mutations in the mtDNA of PBMCs. (*a*) Schematic of human hematopoietic differentiation and lineage commitment. HSC, hematopoietic stem cell; MPP, multipotent progenitor; LMPP, lymphoid-primed multipotent progenitor; CLP, common lymphoid progenitor; CMP, common myeloid progenitor; GMP, granulocyte–monocyte progenitor; MDP, monocyte-dendritic cell progenitor; N CD4, naive CD4^+^ T cell; N CD8, naive CD8^+^ T cell; M CD4, memory CD4^+^ T cell; M CD8, memory CD8^+^ T cell; Th, T helper cell; NK, natural killer cell; pDC, plasmacytoid dendritic cell; Eryth, erythrocyte. (*b*) UMAP projection of 22,312 CD34 + hematopoietic cells and PBMCs with mtscATAC-seq data. Dots represent individual cells, which are colored according to cluster identity. The bar plot indicates the number of cells in each cluster (labeled at right). (*c*) Violin plot showing the number of somatic mtDNA variants per cell for various cell types; *P*-values, two-sided Wilcoxon rank-sum test. (*d*) The VAF distribution of somatic mtDNA mutations across different cell types. Somatic homoplasmic mutations (VAF of ∼1) identified in the lymphoid lineage are highlighted with a red box.

In this study, we focused on the human hematopoietic system, whose cellular differentiation lineages have been well documented. We first identified somatic mtDNA mutations in a previously reported mitochondrial scATAC-seq (or mtscATAC-seq) dataset including more than 20,000 blood cells from a healthy 47-year-old individual (fig. 1*a* and *b*, supplementary fig. S1*a*, Supplementary Material online, see Materials and Methods) (Lareau et al. 2021). We summarized the numbers of mutations and the variant allele frequency (VAF, also referred to as mtDNA heteroplasmic ratio) in each cell to compare the VAF distribution in a population of different cell types. Interestingly, cells of the mature lymphocyte lineages—specifically B, T, and natural killer (NK) cells—had a significantly lower mtDNA mutational burden than hematopoietic progenitor cells, including hematopoietic stem cells (HSCs), multipotent progenitors (MPPs), lymphoid-primed MPPs (LMPPs), common lymphoid progenitors (CLPs), common myeloid progenitors (CMPs), and granulocyte–macrophage progenitors (GMPs) (fig. 1*c*, supplementary fig. S1*b*, Supplementary Material online, Wilcoxon test, *P* < 2.2e^−16^). The mtDNA mutational burden was also lower in lymphocytes than the myeloid and erythroid lineages (fig. 1*c*, Wilcoxon test, *P* < 2.2e^−16^). As anticipated, most somatic mtDNA mutations were detected at low VAF in individual cells in all cell types (fig. 1*d*). However, the distribution of somatic homoplasmic mutations (i.e., those with VAF of ∼1) varied substantially among the different cell types. For instance, progenitor cells, including HSCs, MPPs, LMPPs, CLPs, CMPs, and GMPs exhibited a typical monotonic decline in the number of mutations with increasing VAF (fig. 1*d*). Although this pattern was also seen in both the myeloid and erythroid lineages (e.g., monocytes and erythrocytes), we observed an unanticipated increase in the number of somatic homoplasmic mutations in B, T, and NK cells (fig. 1*d*).

**Fig. 2.**
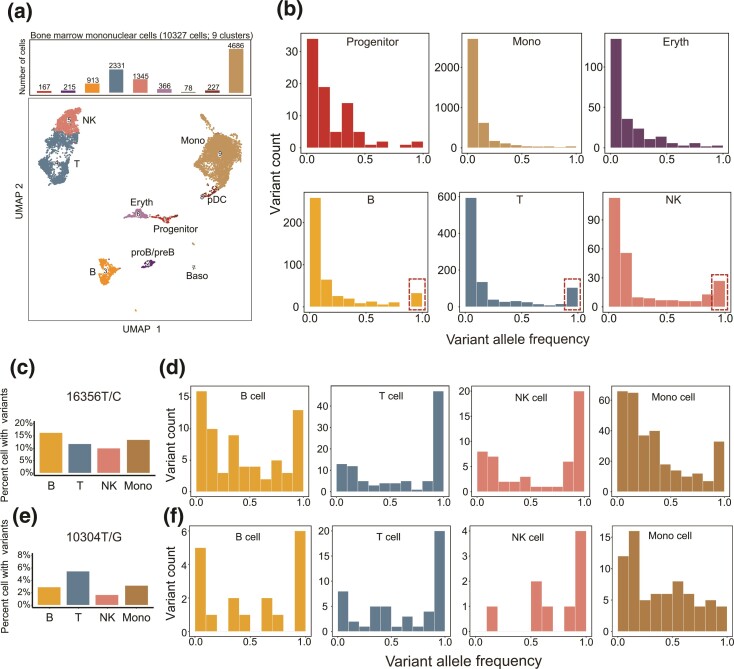
Somatic mutations in the mtDNA of BMMCs. (*a*) UMAP projection of 10,327 mononuclear cells from bone marrow with mtscATAC-seq data. Dots represent individual cells, which are colored according to cluster identify and cell types. (*b*) The VAF distribution of somatic mtDNA mutations across different cell types in BMMCs. Somatic homoplasmic mutations (VAF ∼1) identified in the lymphoid lineage are highlighted with a red box. (*c*) Distribution of the observed mtDNA variant 16356T/C in cells among lymphoid and myeloid cell lineages. (*d*) The VAF distribution of the somatic mtDNA mutation 16356T/C across different cell types. (*e*) Distribution of the observed mtDNA variant 10304G/A in cells among lymphoid and myeloid cell lineages. (*f*) The VAF distribution of the somatic mtDNA mutation 10304G/A across different cell types.

In addition to the mtscATAC-seq dataset from Lareau et al., we analyzed another mtscATAC-seq dataset of 10,327 BMMCs from an independent healthy donor (Mimitou et al. 2021) (fig. 2*a*). Lymphocytes in BMMCs also carried a lower mtDNA mutational burden with a characteristic overrepresentation of somatic homoplasmic mutations (fig. 2*b*, supplementary fig. S1*c* and *d*, Supplementary Material online). Furthermore, to exclude the potential bias of mutations arising at different development time points, we summarized the allele frequency of individual mtDNA mutations shared between lymphoid and myeloid cell lineages. The allele frequency spectrum of the variant 16356T/C and 10304G/A demonstrated that the proportion of cells with homoplasmic variants in B (22% and 33%), T (48% and 40%), and NK (41% and 44%) cells was significantly higher than that in monocytes (10% and 6%) (fig. 2*c*–*f*). In fact, these lymphocyte-specific characteristics were also verified by additional scATAC-seq or scRNA-seq data from seven independent individuals. And the observations were aslo independent of sequencing depth (supplementary figs. S1*e* and S2–S4, Supplementary Material online), thus indicating a general and unique process of mtDNA inheritance in lymphocyte development.

### Asynchronous Replication of Mitochondrial and Nuclear Genomes During B Cell Development

**Fig. 3.**
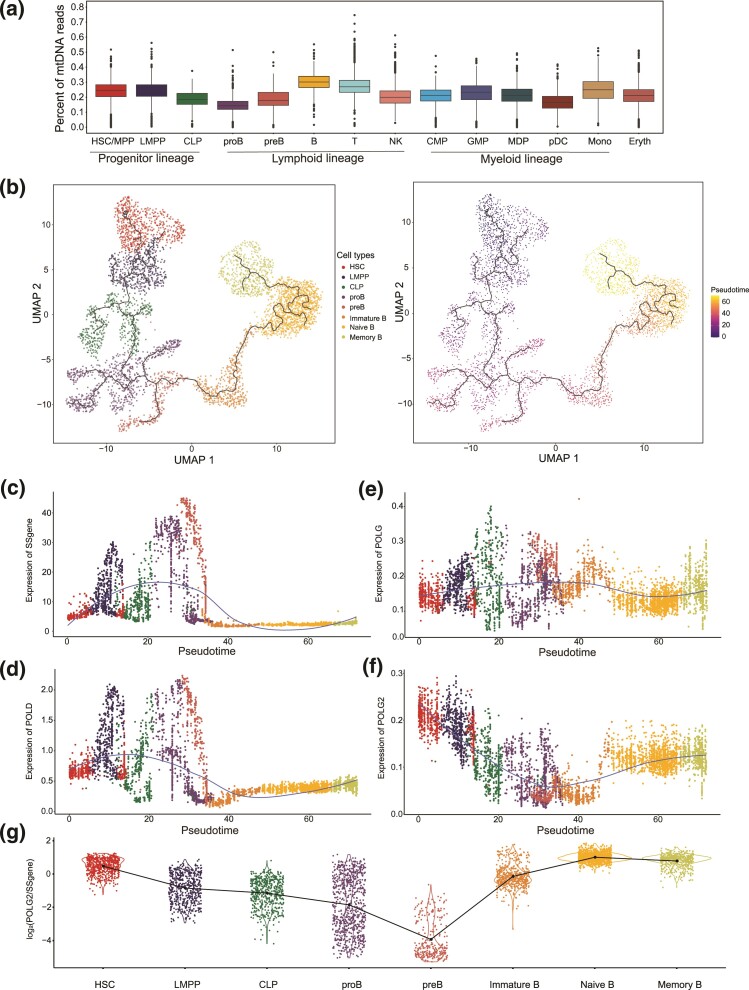
Replication of mtDNA during B cell development. (*a*) The relative number of mtDNA copies was determined according to the proportion (sequencing reads mapped to mitochondrial genome divided by the total number of reads) in each cell, as identified from the scATAC-seq dataset. (*b*) Pseudotime trajectory of B cell differentiation from HSCs by using single-cell RNA-seq data generated from PBMCs (*n* = 4692 cells). Colors denote different cell types (top) and developmental stages (bottom) defined by pseudotime. The solid line represents the fitted trajectory across pseudotime. (*c*–*f*) Kinetic plots showing the expression of (c) 39 G1/S phase-specific genes (SSgene), (*d*) nuclear DNA polymerase δ (*POLD1–3*), (*e*) mtDNA polymerase γ (*POLG*), and (*f*) the binding subunit of mitochondrial DNA polymerase γ (*POLG2*) along the B-cell developmental trajectory. (*g*) Violin plot showing the ratio of *POLG2* expression to the mean expression of all G1/S phase-specific genes in each cell associated with B-cell development. The broken line represents the change trend of the mean ratio across different cell types.

To examine whether the distinct VAF distribution between lymphoid cells and myeloid/erythroid cells was due to the variation in mtDNA copy number per cell, we estimated the relative number of mtDNA copies in each cell type according to the fraction of sequencing reads mapped to the mitochondrial genome relative to the total number of reads in each cell (fig. 3*a*, supplementary fig. S5*a*, Supplementary Material online). Although mature lymphocytes and progenitor cells had similar mtDNA copy numbers, pro-B, and pre-B cells—the earliest lineage-committed cells in B cell development—exhibited a significantly lower number of mtDNA copies (Wilcoxon test, pro-B/pre-B vs. HSC/MPP, *P* < 2.2e^−16^; pro-B/pre-B vs. B, *P* < 2.2e^−16^). Of note, the CLPs also showed significantly fewer mtDNA copies than earlier progenitors did (Wilcoxon test, CLP vs. HSC/MPP, *P* < 2.2e^−16^; CLP vs. LMPP, *P* < 2.2e^−16^), thus indicating a marked mtDNA copy number decrease in early lymphocyte development. Therefore, we hypothesized that the characteristic mutational spectra in lymphocyte mtDNA (figs. 1*c*, *d* and  2*b*) might have resulted from a mitochondrial genetic bottleneck.

To address this possibility, we examined the mtDNA replication machinery to gain insight into the regulation of mtDNA copy number along the lymphocyte differentiation trajectory. We focused on the B cell lineage, because T cells mature in the thymus and their progenitors, pre-T cells, are not present in PBMCs. DNA polymerase γ is the only known mtDNA polymerase in animals (Radsak and Schutz 1978), has both a catalytic (*POLG*) and a binding subunit (*POLG2*), and it catalyzes the polymerization of deoxyribonucleotides. High levels of DNA polymerase γ activity have been detected in the S and G2 cell cycle phases, thereby maintaining stable numbers of mtDNA during cell division (Radsak and Schutz 1978; Chatre and Ricchetti 2013; Sasaki et al. 2017). To determine whether the expression of DNA polymerase γ increases with cell proliferation during B cell development, we projected the developmental trajectory of cell subpopulations from HSCs to mature B cells via a pseudotime analysis with scRNA-seq data (fig. 3*b* and supplementary fig. S5*b*, Supplementary Material online). We observed up-regulation of G1/S phase-specific genes (such as DNA polymerase δ, *POLD1–3*) in both pro-B and pre-B cell populations, thus suggesting high activation of cell proliferation in these cell types (fig. 3*c* and *d*, supplementary fig. S5*c*–*e*, Supplementary Material online). In contrast, the expression of DNA polymerase γ was not coupled with cell proliferation (fig. 3*e* and *f*, supplementary fig. S5*c*–*e*, Supplementary Material online). Unexpectedly, the expression of the DNA polymerase γ binding subunit (*POLG2*) was significantly diminished in the highly proliferative pro-B and pre-B cell subpopulations (fig. 3*g*). Together, these results implied a genetic bottleneck during B cell development which might have resulted from limited replication of mtDNA, thus diluting the mtDNA copy number throughout cell division.

### Quantification of the mtDNA Genetic Bottleneck by Computational Modeling

**Fig. 4.**
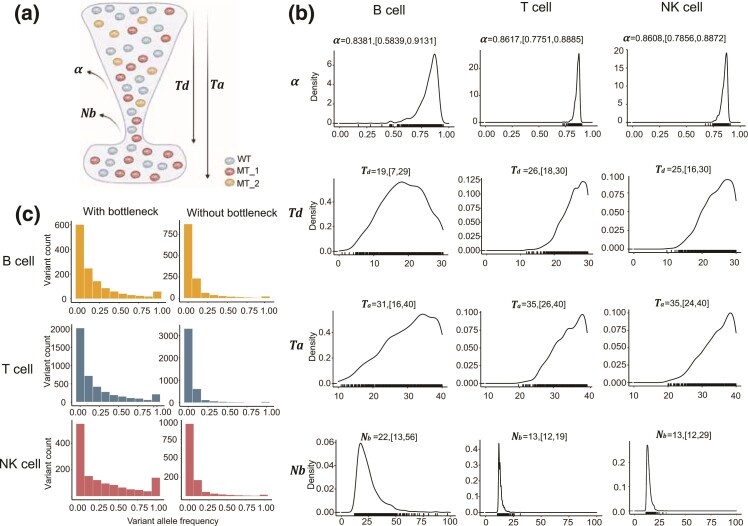
A dilution model of mitochondrial genetic bottleneck. (*a*) Schematic illustration of the dilution model of the mitochondrial genetic bottleneck. In this model, only a fraction of mtDNA molecules (denoted by *α*) replicate at each cell division. After *T_d_* cell divisions from the LMPP stage, the number of mtDNA copies in each lymphocyte subtype (B, T, and NK cells) rapidly recovers to the baseline level (∼500 per cell). *N_b_* denotes the minimal number of mtDNA copies that can be computed as equation (1). The total number of cell divisions required for the transition from LMPP to mature lymphocyte is denoted as *T_a_.* (*b*) The distribution of model parameters inferred by the ABC algorithm. The mean and 95% CI of each parameter estimation are shown. (*c*) Simulations based on the dilution model of mitochondrial genetic bottleneck with the ABC-estimated parameter values recapitulated the lymphocyte-specific overrepresentation of somatic homoplasmic mutations and the lower mutation burden (supplementary fig. S6*b*, Supplementary Material online). The left and right panels represent the simulations with and without mitochondrial genetic bottleneck, respectively. The average of 100 simulations carried out for each model is as shown. The results of each iteration are shown in supplementary fig. S6*c*, Supplementary Material online.

To test whether the genetic bottleneck was achieved by the dilution of mtDNA and quantify the extent of the mitochondrial genetic bottleneck, we developed a computational model of an mtDNA dilution process based on population genetics theory (fig. 4*a*). In this model, we assumed the starting mtDNA copy number is *N*_0_ (∼500 copies per cell estimated by O’Hara et al. 2019) in LMPP cells and only a proportion of mtDNA molecules (denoted by *α*) replicate during each cell cycle. This process of dilution starts from LMPPs and continues for *T_d_* cell generations until the mtDNA copy number recovers to the starting level (∼500 copies). Using the ABC method (see Materials and Methods), we estimated the model parameters for B, T, and NK cell populations by using a constant mtDNA mutation rate of 10^−7^ per site per cell division (fig. 4*b*, supplementary fig. S6*a*, Supplementary Material online) (Coller et al. 2001). The model estimated the minimal mtDNA copy number to be 21 (95% confidence interval [CI] = 13–56), 13 (95% CI = 12–19), and 14 (95% CI = 12–29) in each B, T, and NK cell, respectively. These values were 20–40-fold lower than the normal mtDNA levels. The VAF distribution simulated with these parameter estimations recapitulated the observed data, showing a characteristic overrepresentation of somatic homoplasmic mutations (i.e., VAF of ∼1) and a diminished overall mutational burden (fig. 4*c*, supplementary fig. S6*b* and *c*, Supplementary Material online). Assuming a higher starting mtDNA copy numbers (∼1,000 per cell) resulted in very similar estimations regarding the bottleneck size (supplementary fig. S7*a* and *b*, Supplementary Material online). Therefore, this pattern can not be achieved by random genetic drift with a constant number of mtDNA copies during the lymphocyte development.

### The Consequences of the mtDNA Genetic Bottleneck

**Fig. 5.**
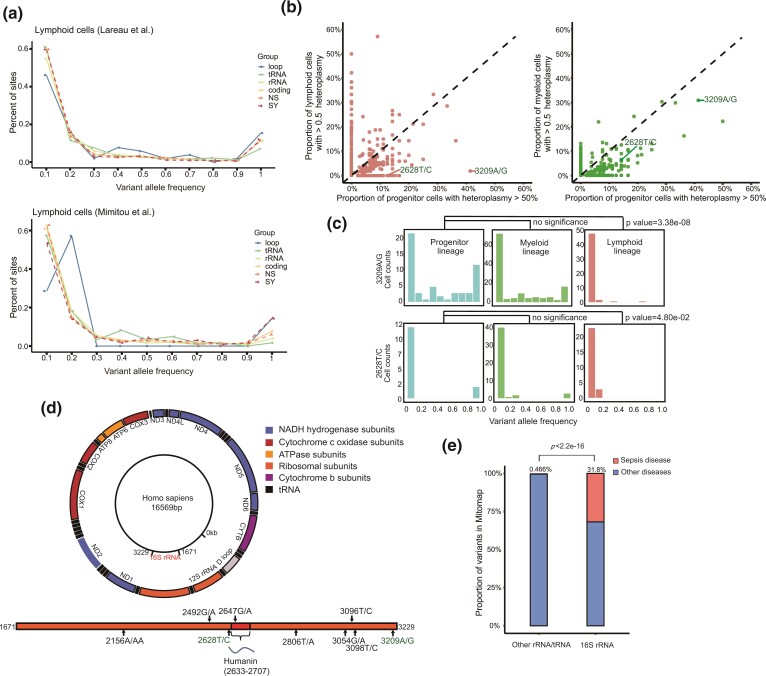
Elimination of specific mtDNA variants in lymphocyte. (*a*) Distribution of the VAF for mutations in different mtDNA genomic regions in lymphoid cells. The color code corresponds to mtDNA genomic regions or mutation types, annotated as loop, tRNA, rRNA, coding (coding region), NS (nonsynonymous), and SY (synonymous). (*b*) Scatter plot showing the percentage of cells with dominant mtDNA mutations (VAF > 50% in a single cell). Results from progenitor cells (HSC, MPP, and LMPP) compared with cells from lymphoid (B, T, or NK cells) or myeloid lineages (right) are shown. (*c*) The distribution of VAF for two individual sites (3209A/G, 2636G/A) in progenitor, myeloid, and lymphoid cells, respectively. The *P*-values shown were determined with the χ^2^ test. (*d*) The location of 16S RNA (MT-RNR2) on the mitochondrial genome and the locations of sepsis associated variants on MT-RNR2, reported in MITOMAP (in black), or those specifically eliminated in lymphocytes (in green). (*e*) The proportion of mtDNA variants associated with sepsis disease in 16S RNA vs. other rRNA/tRNA genes in the mitochondrial genome.

Collectively, our integrative genomic data analysis and computational modeling demonstrated the existence of a stringent mtDNA genetic bottleneck that resulted from replicative dilution during lymphocyte development. This mechanism strengthens the genetic drift toward a lower mtDNA mutational burden and lower genetic diversity within each cell. We wondered whether the genetic bottleneck during lymphocyte development might have the same purifying selection effects as those in the germline. We, therefore, examined the VAF distribution in various genomic regions (loop, tRNA, rRNA, and coding) or mutation types (synonymous [SY] and nonsynonymous [NS]). We found loop and SY regions had higher proportions in somatic homoplasmic mutations than in tRNA, rRNA, and NS regions in datasets (fig. 5*a*, supplementary fig. S8, Supplementary Material online). This result indicated that the mutations in coding regions were more likely to be selected against, when they reached to a high frequency. We next examined the *dN*/*dS* ratio (the ratio of the normalized number of NS substitutions—*dN* to the normalized number of SY substitutions—*dS*) (supplementary fig. S9, Supplementary Material online) according to heteroplasmic categories. The calculated *dN*/*dS* ratios revealed a pattern of generally neutral evolution (i.e., *dN*/*dS* ∼ 1) in all categories in most of the cases examined (supplementary fig. S9, Supplementary Material online). The *dN*/*dS* ratio might not be sensitive in testing selection in somatic cells, owing to the linkage of whole mitochondrial genome with strong Hill–Robertson interference, thus leading to a pattern of quasi-neutrality, as in cancer evolution (Chen et al. 2019). Therefore, we verified individual mutation sites to identify the signals of purifying selection; indeed, we observed several mutations that were specifically eliminated in lymphocytes but not the myeloid lineage (fig. 5*b*). For example, the mutations, 2636G/A, and 3209A/G, underwent the most profound decrease in prevalence (fig. 5*c*) in lymphocytes. Intriguingly, these two sites are all located at MT-RNR2, which encodes 16S rRNA and Humanin, a peptide protective against multiple mitochondrial diseases (fig. 5*d*) (Lee et al. 2013). Furthermore, we queried MITOMAP, a human mitochondrial genome database, and found that mtDNA variants reported on MT-RNR2 were highly associated with sepsis (Wilcoxon test, *P* < 2.2e^−16^, fig. 5*e*) (Lott et al. 2013; Park et al. 2021); therefore, MT-RNR2 may play important roles in immune functions protecting against infections. These data indicated that purifying selection in lymphocytes indeed occurs for specific mtDNA mutation sites.

## Discussion

Collectively, our results indicated an unanticipated lower mutational burden and accumulation of homoplasmic mtDNA mutations in lymphocytes, thus indicating a stringent genetic bottleneck and purifying selection of mtDNA. Gene expression data and computational modeling suggested a dilution process, based on the rate of mtDNA replication relative to the nuclear genome.

The single-cell data derived from PBMCs could not capture the full developmental trajectory of T cells because pre-T cells develop in the thymus. Our single-cell data and computational inference indicated that the genetic bottleneck in T and NK cells might be as stringent as that in B cells (figs. 1*d*, 2*a*, and  4*b*). On the basis of our observations and simulations, we hypothesized that the regulation of the lymphocyte-specific genetic bottleneck may start from the CLP stage, instead of subsequent lineage commitment for B, T, and NK cells. The effect of this regulation is likely to be enhanced via the active proliferation of progenitor cells. During lymphocyte development, multipotent T and B progenitor cells undergo a series of maturation steps that include positive selection for functional T-cell receptors or immunoglobulins and negative selection to eliminate cells with a high affinity toward self-associated peptides or antigens (Starr et al. 2003). Only a small proportion of T lymphoid cells will survive after the negative and positive selections. Moreover, mitochondrial function is important for T cell development and their functional activation (Buck et al. 2016; Chao et al. 2017). The metabolic responses characteristic of lymphocytes development and activation are both well-regulated at the transcriptional and posttranscriptional levels (Maciver et al. 2013). For example, several groups have shown that T or B cell activation leads to mitochondrial remodeling and dramatic shifts in cell metabolism, in the course of their role in eliminating pathogens (Sena et al. 2013; West and Shadel 2017; Angajala et al. 2018; Waters et al. 2018; Lee et al. 2022). Furthermore, the selection against the pathogenic mutations 3243 was stronger in T cells than in B and NK cells, as shown by Walker et al. (2020). Further systematic study of T precursors in thymus will provide further insight into how genetic bottlenecks occur during T cell development.

On the basis of the scRNA-seq data, we found higher expression of Drp1 and LC3 genes in pro/pre-B cells than the other progenitor cells, thus implying that mitochondria at this stage may undergo fission or mitophagy process (Lemasters 2005). Further experimental evidence will be helpful to determine whether mitophagy of mitochondria is also involved in the genetic bottleneck process in the progenitor cell stage. If so, the selection of mtDNA mutations might also happen in mitochondrial organelles level.

Our computational framework modeled a single-cell lineage and quantitatively measured the mtDNA bottleneck size after the continual dilution process. Because B/T/NK cell development and maturation are complicated process involving a series of clonal selections on cellular levels, such as positive and negative selection of pre-B/pre-T cells in the primary lymphoid organs (Starr et al. 2003), we were unlikely to model all those selection processes on specific cell types within our model. Therefore, to make our model simple and robust, we only considered a neutral process of continual dilution with mtDNA copy number changes at the single-cell level. Furthermore, the mutation rate in our model is sensitive to the detection of low-frequency mutations. Ultradeep sequencing of the mitochondrial genome through other single-cell mtDNA sequencing methods (Guo et al. 2020) would aid in estimating the mutation rate accurately and would further facilitate the estimation of selection strength.

Our newly discovered somatic mtDNA bottleneck within the lymphoid lineage may play a role in the quality control of mitochondrial genomes, in parallel to the selection of immunoreceptor genes in the nuclear genome. Thus, a robust population of mtDNA may be crucial for lymphocyte-mediated immune responses. This mtDNA genetic bottleneck may be one of several potential mechanisms involved in the regulation of mitochondrial genome in different lineages. Currently, there is not available for high-quality mtscATAC datasets in many different cell types, it is unknown whether the stringent mtDNA genetic bottleneck is a lymphocyte-specific phenomenon or also prevalent in other cell types. Hence, the prevalence, causes, and the consequences of the somatic mtDNA genetic bottleneck require extensive exploration in future studies. The understanding of the mtDNA quality control mechanism may ultimately provide new insights into immune degeneration and related diseases, and contribute to the treatments based on engineered immune cells, such as chimeric antigens receptor-T cell therapy.

## Materials and Methods

### Data Collection

The mtscATAC-seq dataset generated through evaluation of hematopoietic and PBMCs was retrieved from a recent study evaluating samples from a healthy 47-year-old donor (Lareau et al. 2021). The mtscATAC-seq dataset from human bone marrow from a healthy 25-year-old donor was obtained from Mimitou et al. (2021). The scATAC-seq data from CD4^+^ T cells were obtained from the study published by Satpathy et al (2018). The scATAC-seq dataset for HSCs, MPPs, LMPPs, CLPs, CMPs, GMPs, and plasmacytoid dendritic cells (pDCs) derived from CD34^+^ bone marrow was obtained from Buenrostro et al (2018) (supplementary fig. S2, Supplementary Material online). The scRNA-seq dataset generated from an evaluation of healthy CD34^+^ PBMCs, BMMCs, and total PBMCs was downloaded from a study published by Granja et al. (2019). These datasets were used to analyze mtDNA replication and gene transcription. The scRNA-seq dataset of 70 effector memory T cells (Tem cells), 70 central memory T cells (Tcm cells), and 142 CD4^+^ regulatory T cells (Treg cells) from healthy human colon tissue were downloaded from Array Express (E-MTAB-6072) (Miragaia et al. 2019). Detailed information on data resources is provided in supplementary table S1, Supplementary Material online.

### Single-Cell (sc)ATAC-seq Data Preprocessing and Annotation of the Cell Populations

Raw data from GSE142745 were processed with Cell Ranger ATAC (version 2.0.3; 10× Genomics, https://www.10xgenomics.com/products/single-cell-atac) with default parameters. Reads were aligned to the reference hg19 human genome (https://support.10xgenomics.com/single-cell-atac/software/downloads/latest). In each cell, 40% of fragments overlapping a compendium of DNase hypersensitivity peaks and 1,000 unique nuclear fragments were filtered. From the output of the Cell Ranger Software calls, we performed a computational annotation of the cell types on the basis of chromatin accessibility. Clustering and gene activity scores were determined through standard processing via ArchR (Granja et al. 2021). Clustering was performed with the “addClusters” and “addUMAP” functions (resolution = 0.8, neighbors = 10, minDist = 0.1). To identify marker genes according to gene scores, we used the “getMarkerFeatures” function with useMatrix “GeneScoreMatrix” and generated a reproducible peak set in ArchR by using the “addReproduciblePeakSet” function. By default, ArchR attempts to identify peaks by using the MACS2 algorithm (Zhang et al. 2008). Because common cell markers are sometimes not suitable for classification with “GeneScoreMatrix,” we used enhancer accessibility to define the cell type. For example, we identified myeloid cells according to the unique accessibility of enhancers at +85 and +87 kb in the interferon regulatory factor (*IRF8*) locus. pDCs were identified on the basis of the unique accessibility of +54 and +56 kb enhancers, as described by Satpathy et al (2018). Furthermore, to label scATAC-seq clusters with scRNA-seq information, we used the “addGeneIntegrationMatrix” function, which integrates scATAC-seq with scRNA-seq. Specific marker genes used to identify individual cell types in scATAC-seq datasets of healthy CD34^+^ hematopoietic cells and PBMCs are documented in supplementary table S2, Supplementary Material online.

### mtDNA Variants Identified in Single-Cell ATAC-seq Datasets

Paired-end raw reads from each sample were aligned to the human reference genome (hg19) with Cell Ranger ATAC after adapter sequences were trimmed. First, the reads mapped to multiple sites or the nuclear genome, and duplicates were also removed. The remaining reads were realigned to correct the potential mapping errors around indels according to the process from GATK (McKenna et al. 2010). Bam files for each cell type were merged to identify germline mtDNA variants (bulk VAF > 90%). Variants with VAF > 90% shared among more than 90% cells were also considered germline mutations. Then mtDNA variants were called for each individual cell with VarScan2 (Koboldt et al. 2012) with “–min-var-freq 0.01,” “min-depth 8,” and “–min-reads2 2” at the beginning. To identify high confidence somatic variants in single cell, we applied the following filtering steps.

First, the germline mutations identified in the merged bam file were removed. Second, the following sites were explicitly removed because of the large numbers of homopolymers in the revised Cambridge Reference Sequence (rCRS) and sequencing errors in the reference genome (Ju et al. 2014): (1) misalignment due to ACCCCCCCTCCCCC (rCRS 302–315), including 302A/C, 309C/T, 311C/T, 312C/T, 313C/T, and 316G/C; (2) misalignment due to GCACACACACACC (rCRS 513–525), including 514C/A, 515A/G, 523A/C, and 524C/G; (3) misalignment due to 3107N in ACNTT (rCRS 3105–3109), including 3106C/A, 3109T/C, and 3110C/A. Third, sequencing errors can significantly affect the identification of somatic variants. Therefore, sequencing errors known to be associated with a high error rate according to Illumina NextSeq and sequence errors (G → T and C → A) from DNA damage were removed. Fourth, each mtDNA site count was required to be more than 20 reads (20×), and the variant count more than two reads. Fifth, strand balance was required for confident somatic variants. For the given variant site, we required the reads mapped to the forward strand to be above 30% but below 70% of the total mapped reads for the variant allele. Variants that passed the multiple filter steps were merged from all individual cells as the final somatic variants. If the findings for a variant had sufficient confidence in any given cell, the VAF was recounted in all individual cells within the same cell type, with constraints of a minimum 8× depth. Cell with an average depth more than 10× were considered for further analysis.

### Single-Cell RNA-seq Data Processing and Cell-Type Annotation

Downstream analysis of the scRNA-seq dataset was performed with Seurat (Stuart et al. 2019) (version 3.2.2; https://satijalab.org/seurat). The following bioinformatic analyses were performed in R software (version 3.6.0; https://www.r-project.org) with default settings unless otherwise stated. Cells with <200 or >2,500 detected genes or with >5% mtDNA were eliminated from further consideration. Normalization was applied with the MAGIC package (version 2.0.3) (van Dijk et al. 2018) by following the Seurat v3 workflow. We next calculated a subset of features that exhibited high cell-to-cell variability by using the “FindVariableFeatures” function and identified 2,000 specific features. Clusters were identified with the “Find-Neighbors” and “FindClusters” functions in Seurat with 45 principal components (PCs) and a resolution of 0.3. The results were annotated to include differential expression of cell type-specific marker genes. Uniform Manifold Approximation and Projection (UMAP) for dimensionality reduction was performed with the “RunUMAP” function in Seurat, with 45 PCs and other default parameters. The expression of cell type-specific marker genes in PBMCs and BMMCs is shown in supplementary table S3, Supplementary Material online. We referred to the information and classifications recorded in GSE139369 from the GEO database to guide our cell type annotations (supplementary table S3, Supplementary Material online).

### Pseudotime Analysis

To construct single-cell differentiation trajectories with scRNA-seq data from HSCs to B cells, we performed a pseudotime analysis with the Monocle method (Trapnell et al. 2014; Qiu et al. 2017; Cao et al. 2019). First, we subdivided scRNA-seq data according to the annotated cell populations revealed by Seurat clustering analysis, according to the common pipeline (http://cole-trapnell-lab.github.io/monocle-release/monocle3/). Reclustering of selected cell populations was again performed with the “RunUMAP” function. Pseudotime analysis was conducted on these newly generated clusters with Monocle v3. We delineated expression patterns of G1/S phase-specific and mtDNA replication-related genes along a pseudotimeline. G1/S phase-specific genes were identified according to a previously annotated list (supplementary fig. S5*d*, Supplementary Material online) (Tirosh et al. 2016).

### mtDNA Variants Identified from Single-Cell RNA-seq Data

mtDNA variants from single-cell RNA-seq data were processed in the same manner as mtDNA variants from scATAC-seq, with several modifications. Briefly, we used STAR (Dobin et al. 2013) to align reads to the human reference genome (hg19) and to obtain bam files. Germline mutations and mtDNA variants in individual cells were filtered and called in the same manner.

### Allele Frequency Spectrum

The allele frequency (heteroplasmic ratio) of each mutation was calculated in each cell and the number of mutations that fall in each frequency bin (from 0 to 1) was counted for each cell type. Somatic mutations that arose in the early development stage, which had been fixed in the progenitor cells, were further excluded for the AFS analysis in the mtscATAC-seq on BMMCs.

### Annotation of Mitochondria DNA Mutations and Calculation of NS/SY Mutation Rates (*dN*/*dS*)

The mitochondrial variants were annotated with ANNOVAR (Wang et al. 2010). The annotated variants comprised mutations in loops, tRNA, rRNA, and mRNA coding regions, including NS and SY substitutions according to the variant location (supplementary figs. S8 and S9, Supplementary Material online). Coding sequences (CDS) within the mitochondrial genome were evaluated with Phylogenetic Analysis of Maximum Likelihood to identify all possible SY (defined as *S*) and NS (defined as *N*) substitutions in the human mitochondrial genome (Yang 2007). On the basis of ANNOVAR’s annotations, we identified all observed SY (defined as *s*) and NS substitutions (defined as *n*). The NS mutation rate (*dN*) = *n*/*N* and the SY mutation rate (*dS*) = *s*/*S*, responses to positive, neutral, or negative selection pressure, can be determined by the *dN*/*dS* ratio.

### Computational Modeling of the Mitochondrial Genetic Bottleneck

We used the Wright-Fisher model from population genetics to depict the accumulation of mutations and the dynamic frequency of heteroplasmic alleles in mtDNA during lymphoid cell divisions. The Wright-Fisher model assumes discrete generations and random sampling of individuals from the current generation without replacement by reproduction in the following generation. This model has been widely used to model the mtDNA population dynamics in both germline cells and somatic cells, including those that are neoplastic (Coller et al. 2001; Wilton et al. 2018). Because normal somatic cells typically contain 100–1,000 copies of mtDNA, we used *n* = 500 as the baseline copy number in our model (O’Hara et al. 2019). Results from the scATAC dataset revealed that the relative copy number of mtDNA in NK cells was ∼60% that detected in B or T cells (fig. 3*a*); thus, 300 (500 × 0.6) was used as the baseline mtDNA copy number for the NK lymphocyte cohort. We modeled the lymphoid development from LMPP cells, which are the common progenitor cells for all lymphocytes, B, T, and NK cells. To model the dilution-based genetic bottleneck, we introduced a dilution rate *α*, which denotes the fraction of mtDNA molecules in each cell that undergo replication within a single-cell cycle, and *T_d_*, which denotes the time of the diluting process. After *T_d_* cell divisions from LMPP, the mtDNA copy number in each cell type rapidly recovers to the baseline level. The minimal mtDNA copy number through the bottleneck can be computed by:

(1) $$N_{b}=N_{0}\alpha^{T_{d}}$$

where *N*_0_ is the initial number of mtDNA copies. The total number of cell divisions required for the transition from an LMPP to a mature lymphocyte is denoted *T_a_*. The mutation rate at each site within the mitochondrial genome per cell division is denoted *u*, which has been estimated to be 10^−8^ to 10^−7^ mutations per site for somatic cells (Coller et al. 2001; Cabrera 2021). Thus, the mutation rate for the entire mitochondrial genome during each cell division event will be *u* = *μ*  × *L*, where *L* = 16,569 base pairs (bp), representing the number of potential sites within the mtDNA length.

During each cell division, the number of somatic mutations acquired per mitochondrial genome follows a Poisson distribution with a mean of *u*. Thus, the probability that *k* mutations occurred in each cell division is as follows:

(2) $$P(x=k)=\frac{u^{k}e^{-u}}{k!}$$

### Computational Inference of Parameters by ABC

We used the framework of ABC for parameter inference in our computational model of somatic mtDNA population dynamics on the basis of the dilution rate *α*, the dilution time course *T_d_* and the total number of cell divisions *T_a_*. The minimal mtDNA copy number in each cell can be computed as described by equation (1) when values for *α* and *T_d_* are available. The prior uniform distributions used for sampling *α*, *T_d_*, and *T_a_*, were, $T_{d}∼U(0,\;30)$ and $T_{a}∼U(10,\;40)$. To avoid extinction (i.e., a minimal mtDNA copy number of 0), only the sampled parameter values ensuring $N_{b}\;(=N_{0}\alpha^{T_{d}})>10$ were retained. We used a version of ABC based on the acceptance–rejection algorithm (Tavare et al. 1997) to estimate posterior probability distributions for the parameters of interest (i.e., ***θ***, *T*_*d*_). We used 19 summary statistics (***S***), which included the mtDNA mutation count in each VAF with a step size of 0.05 from VAF = 0.05 to 1 to fit the simulated to the observed data. The ABC version of rejection sampling is as follows:

1. Sample parameters *θ*^′^ from the prior distribution *π* (**θ**)
2. Simulate data (***D***^′^) with the sampled parameters (*θ*^′^) and summarize ***D***^′^ as summary statistics (***S***^′^)
3. Accept *θ*^′^ if *d*(***S***^′^, ***S***) < *ε*, for a given tolerance rate *ε*, where *d*(***S***^′^, ***S***) is a measure of the Euclidean distance between ***S***^′^ and ***S***
4. Return to step 1.

With this scheme, we approximated the posterior distribution by *P*(***θ***| *d*(***S***^′^, ***S***) < *ε*). We used a common variation in ABC (Beaumont et al. 2002; Zhao et al. 2014) in which, rather than using a fixed threshold, *ε*, we sorted all calculated *K* distances by *d*(***S***^′^, ***S***) (see step 3 above) and accepted the *θ*^′^ that generated the smallest 100 × *η* percentage distances. We used *K* = 10^6^ and *η* = 0.001 so that the posterior distribution was composed of 10^6^ × 0.001 = 1,000 data points. We ran the ABC inference procedures for two mutation rates (*μ* = 10^−8^ and 10^−7^) and performed model selection (supplementary fig. S6, Supplementary Material online). The mutation rate *μ* = 10^−7^ fitted the data better in all cell types and thus was used for the computational inference. The ABC procedure was performed with the R package *abc* (Csilléry et al. 2012).

## Supplementary Material

Supplementary data are available at *Molecular Biology and Evolution* online.

## Supplementary Material

msac090_Supplementary_Data

## Data Availability

Detailed information on data resources is provided in supplementary table S1, Supplementary Material online. Code used for single-cell data analysis and computational modeling are available at https://github.com/tangzhj/Bottleneck. ***Conflict of Interest.*** H.Y.C. is a co-founder of Accent Therapeutics, Boundless Bio, Cartography Biosciences, and is an advisor to 10x Genomics, Arsenal Biosciences, and Spring Discovery. The other authors declare no conflicts of interest.
