# Supplementary material for: A Genetic Bottleneck of Mitochondrial DNA During Human Lymphocyte Development
Source: Mol Biol Evol. 2022 Apr 28;39(5):msac090. doi: 10.1093/molbev/msac090 (PMC9113143; doi:10.1093/molbev/msac090)
Supplement: msac090_Supplementary_Data [file msac090_supplementary_data.zip › Supplementary_Figures.pdf]

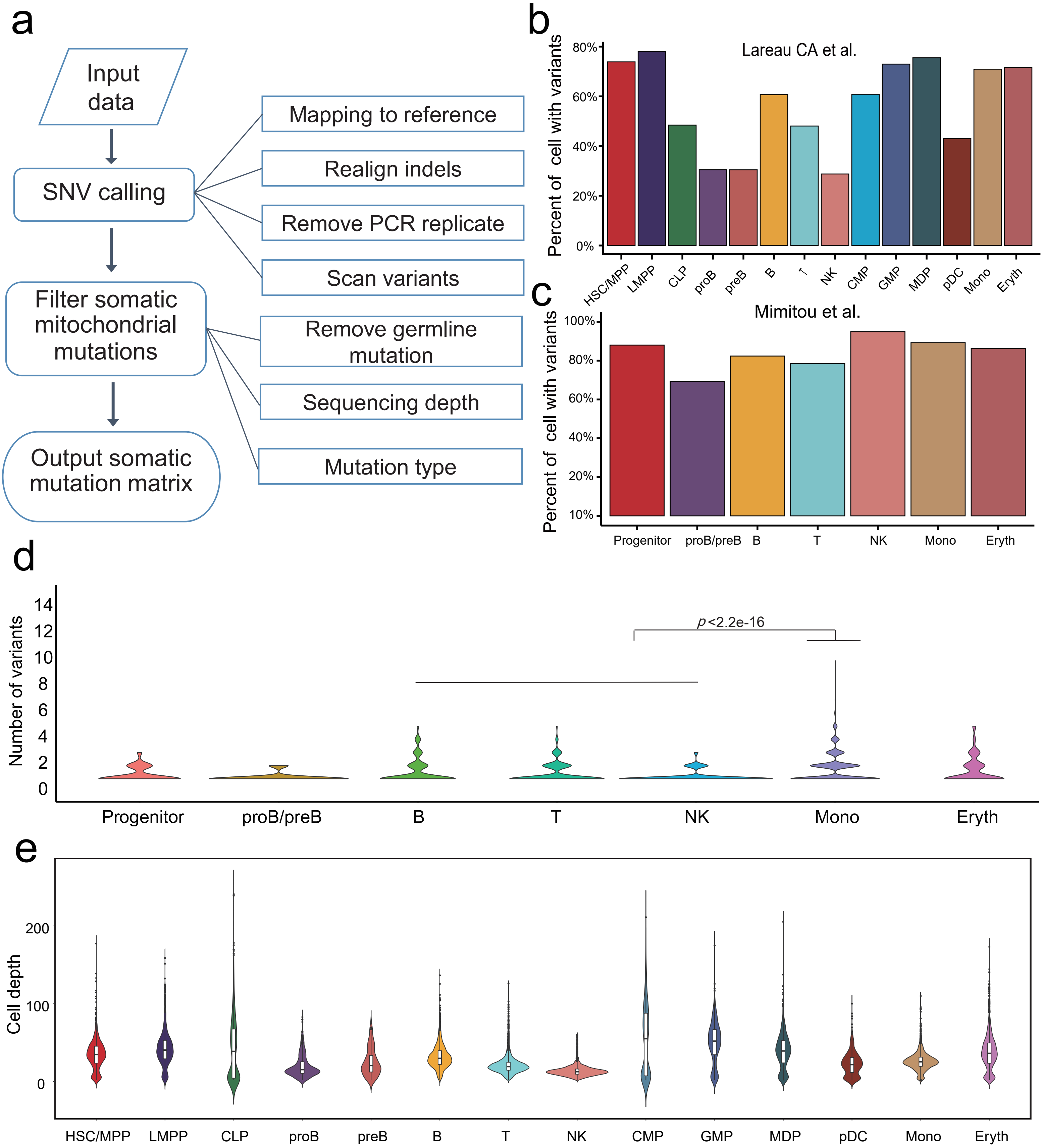

a
Input data
SNV calling
Filter somatic mitochondrial mutations
Output somatic mutation matrix
Mapping to reference
Realign indels
Remove PCR replicate
Scan variants
Remove germline mutation
Sequencing depth
Mutation type
b
Lareau CA et al.
Percent of cell with variants
80%
60%
40%
20%
0%
HSC/MPP
LMPP
CLP
proB
preB
B
T
NK
CMP
GMP
MDP
pDC
Mono
Eryth
c
Mimitou et al.
Percent of cell with variants
100%
80%
60%
40%
20%
10%
Progenitor
proB/preB
B
T
NK
Mono
Eryth
d
Number of variants
14
12
10
8
6
4
2
0
p<2.2e-16
Progenitor
proB/preB
B
T
NK
Mono
Eryth
e
Cell depth
200
100
0
HSC/MPP
LMPP
CLP
proB
preB
B
T
NK
CMP
GMP
MDP
pDC
Mono
Eryth

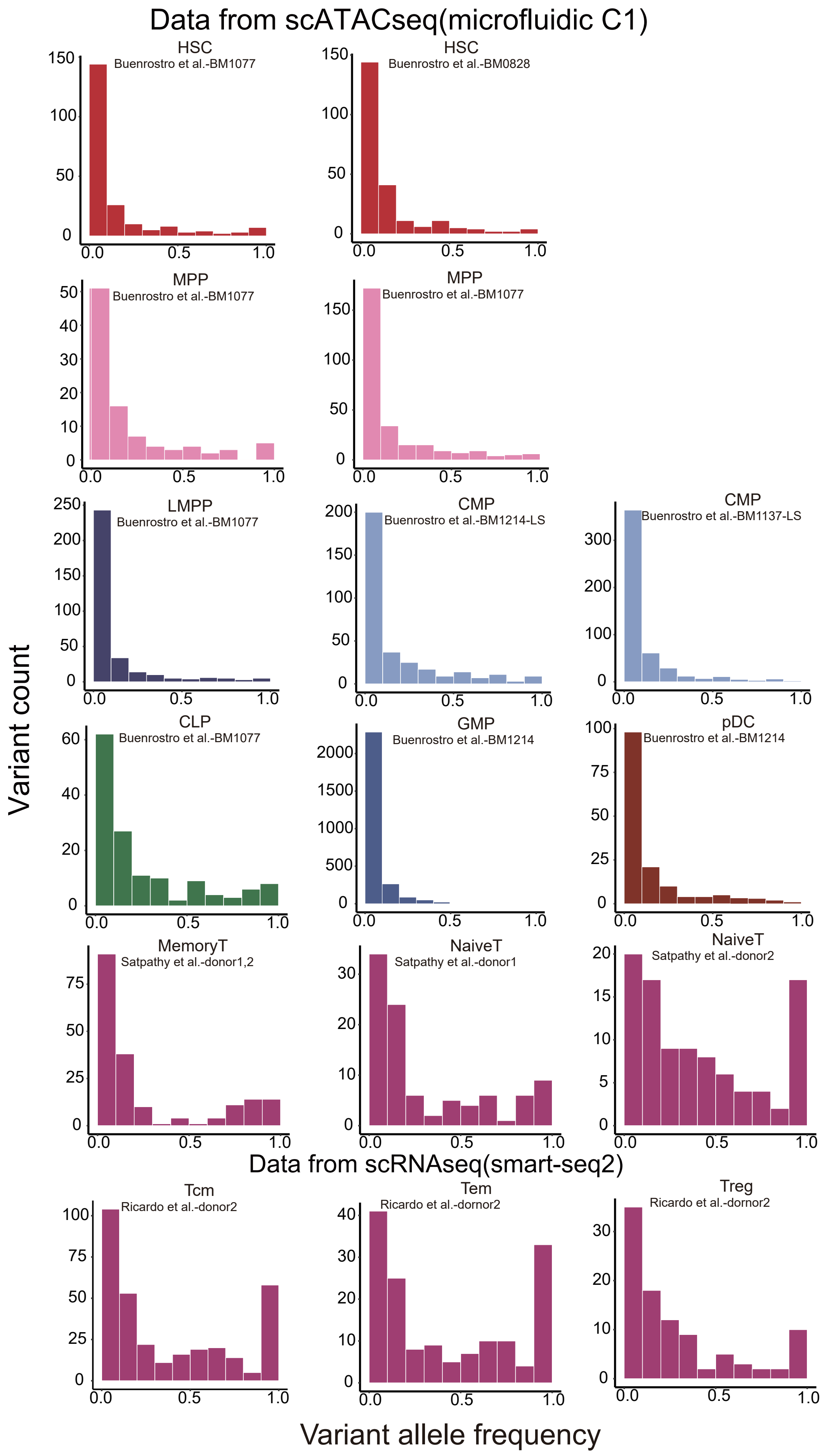

Data from scATACseq(microfluidic C1)
HSC
Buenrostro et al.-BM1077
HSC
Buenrostro et al.-BM0828
MPP
Buenrostro et al.-BM1077
MPP
Buenrostro et al.-BM1077
LMPP
Buenrostro et al.-BM1077
CMP
Buenrostro et al.-BM1214-LS
CMP
Buenrostro et al.-BM1137-LS
CLP
Buenrostro et al.-BM1077
GMP
Buenrostro et al.-BM1214
pDC
Buenrostro et al.-BM1214
MemoryT
Satpathy et al.-donor1,2
NaiveT
Satpathy et al.-donor1
NaiveT
Satpathy et al.-donor2
Data from scRNAseq(smart-seq2)
Tcm
Ricardo et al.-donor2
Tem
Ricardo et al.-dornor2
Treg
Ricardo et al.-dornor2
Variant count
Variant allele frequency

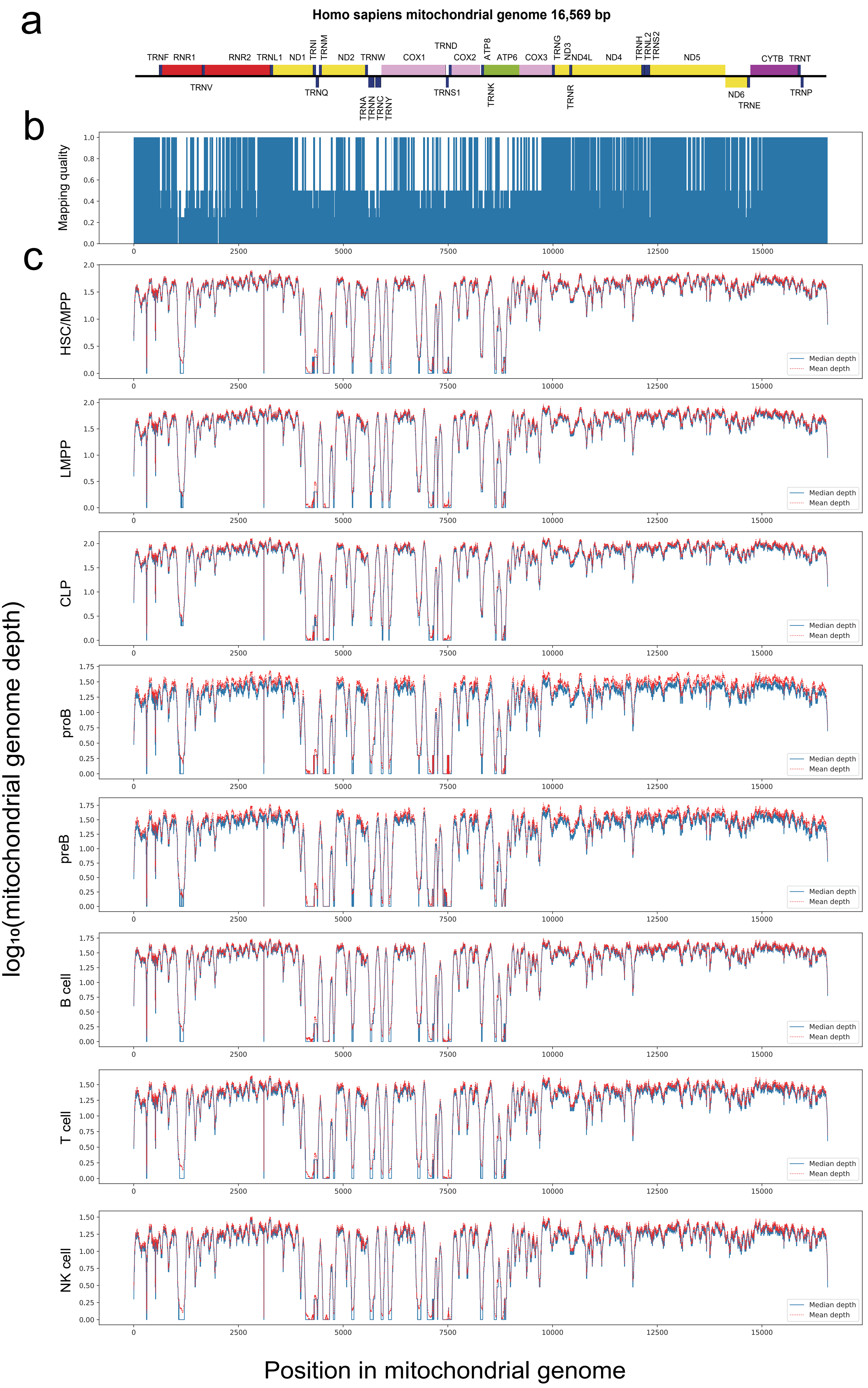
a
Homo sapiens mitochondrial genome 16,569 bp
TRNF RNR1 RNR2 TRNL1 ND1 TRNI TRNM ND2 TRNW COX1 TRND COX2 ATP8 ATP6 COX3 TRNG ND3 ND4L ND4 TRNH TRNL2 TRNS2 ND5 CYTB TRNT
TRNV TRNQ TRNA TRNN TRNC TRNY TRNS1 TRNK TRNR ND6 TRNE TRNP
b
Mapping quality
c
HSC/MPP
LMPP
CLP
proB
preB
B cell
T cell
NK cell
Median depth
Mean depth
log10(mitochondrial genome depth)
Position in mitochondrial genome

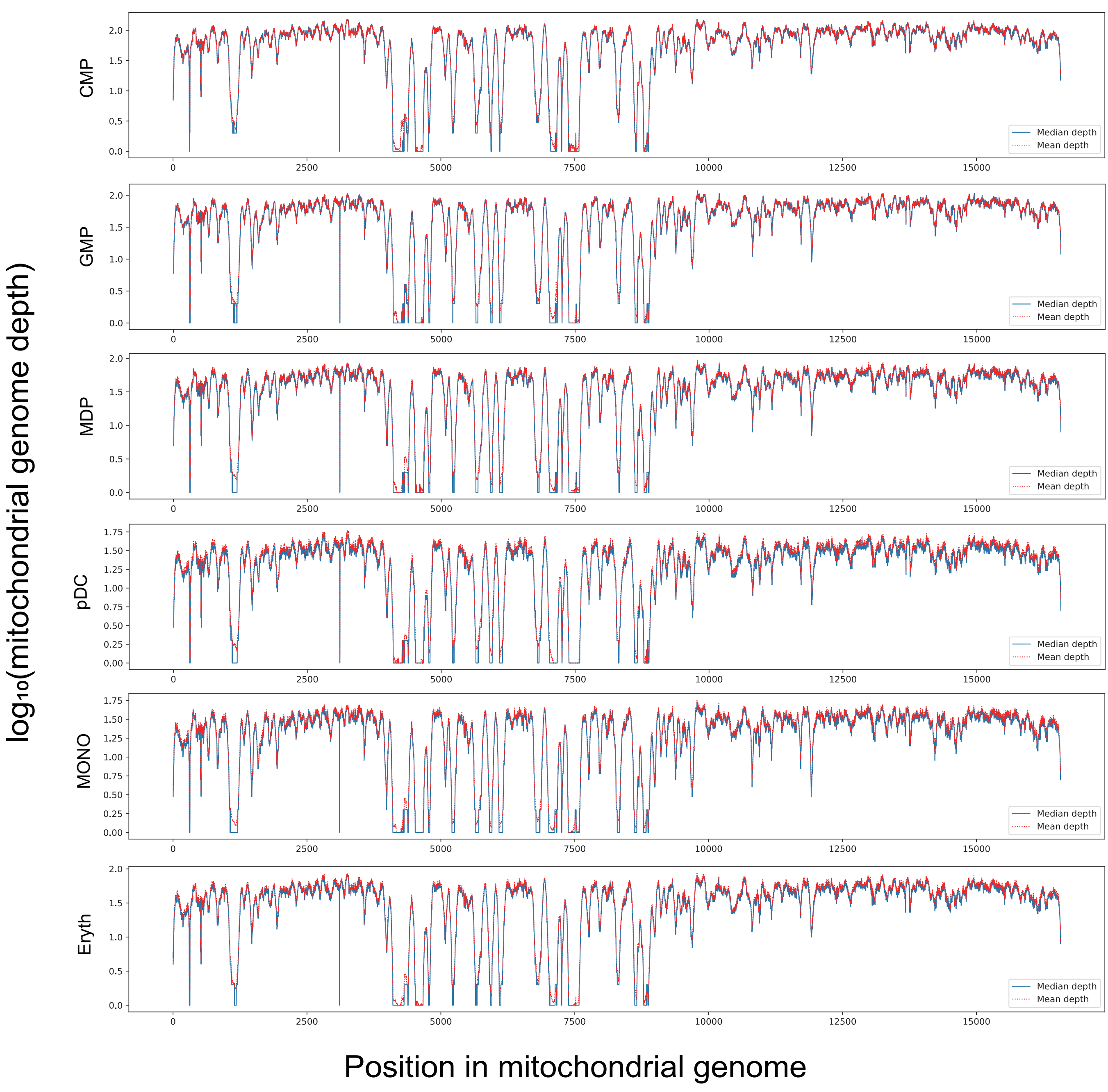
log₁₀(mitochondrial genome depth)
CMP
GMP
MDP
pDC
MONO
Eryth
Median depth
Mean depth
Position in mitochondrial genome

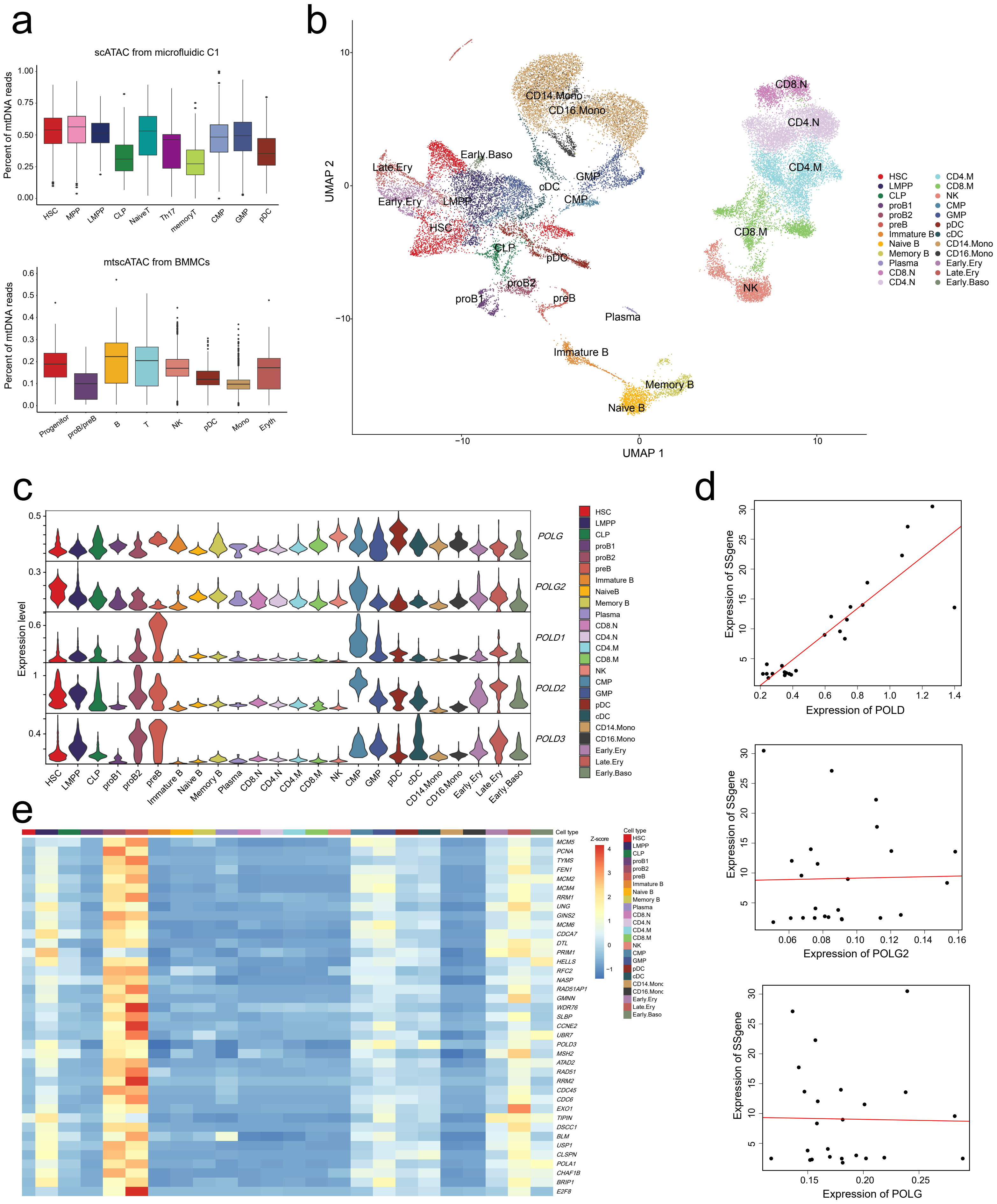
a
scATAC from microfluidic C1
Percent of mtDNA reads
1.00
0.75
0.50
0.25
0.00
HSC
MPP
LMPP
CLP
NaiveT
Th17
memoryT
CMP
GMP
pDC
mtscATAC from BMMCs
Percent of mtDNA reads
0.6
0.5
0.4
0.3
0.2
0.1
0.0
Progenitor
proB/preB
B
T
NK
pDC
Mono
Eryth
b
UMAP 2
UMAP 1
10
0
−10
CD14.Mono
CD16.Mono
Early.Baso
Late.Ery
Early.Ery
LMPP
HSC
GMP
cDC
CMP
CLP
pDC
proB2
proB1
preB
Plasma
Immature B
Memory B
Naive B
CD8.N
CD4.N
CD4.M
CD8.M
NK
HSC
LMPP
CLP
proB1
proB2
preB
Immature B
Naive B
Memory B
Plasma
CD8.N
CD4.N
CD4.M
CD8.M
NK
CMP
GMP
pDC
cDC
CD14.Mono
CD16.Mono
Early.Ery
Late.Ery
Early.Baso
c
Expression level
0.5
0.3
0.6
1
0.4
POLG
POLG2
POLD1
POLD2
POLD3
HSC
LMPP
CLP
proB1
proB2
preB
Immature B
Naive B
Memory B
Plasma
CD8.N
CD4.N
CD4.M
CD8.M
NK
CMP
GMP
pDC
cDC
CD14.Mono
CD16.Mono
Early.Ery
Late.Ery
Early.Baso
HSC
LMPP
CLP
proB1
proB2
preB
Immature B
NaiveB
Memory B
Plasma
CD8.N
CD4.N
CD4.M
CD8.M
NK
CMP
GMP
pDC
cDC
CD14.Mono
CD16.Mono
Early.Ery
Late.Ery
Early.Baso
d
Expression of SSgene
Expression of POLD
30
25
20
15
10
5
0.2
0.4
0.6
0.8
1.0
1.2
1.4
Expression of SSgene
Expression of POLG2
0.06
0.08
0.10
0.12
0.14
0.16
Expression of SSgene
Expression of POLG
0.15
0.20
0.25
e
Cell type
Z-score
4
3
2
1
0
−1
Cell type
HSC
LMPP
CLP
proB1
proB2
preB
Immature B
Naive B
Memory B
Plasma
CD8.N
CD4.N
CD4.M
CD8.M
NK
CMP
GMP
pDC
cDC
CD14.Monc
CD16.Monc
Early.Ery
Late.Ery
Early.Baso
MCM5
PCNA
TYMS
FEN1
MCM2
MCM4
RRM1
UNG
GINS2
MCM6
CDCA7
DTL
PRIM1
HELLS
RFC2
NASP
RAD51AP1
GMNN
WDR76
SLBP
CCNE2
UBR7
POLD3
MSH2
ATAD2
RAD51
RRM2
CDC45
CDC6
EXO1
TIPIN
DSCC1
BLM
USP1
CLSPN
POLA1
CHAF1B
BRIP1
E2F8

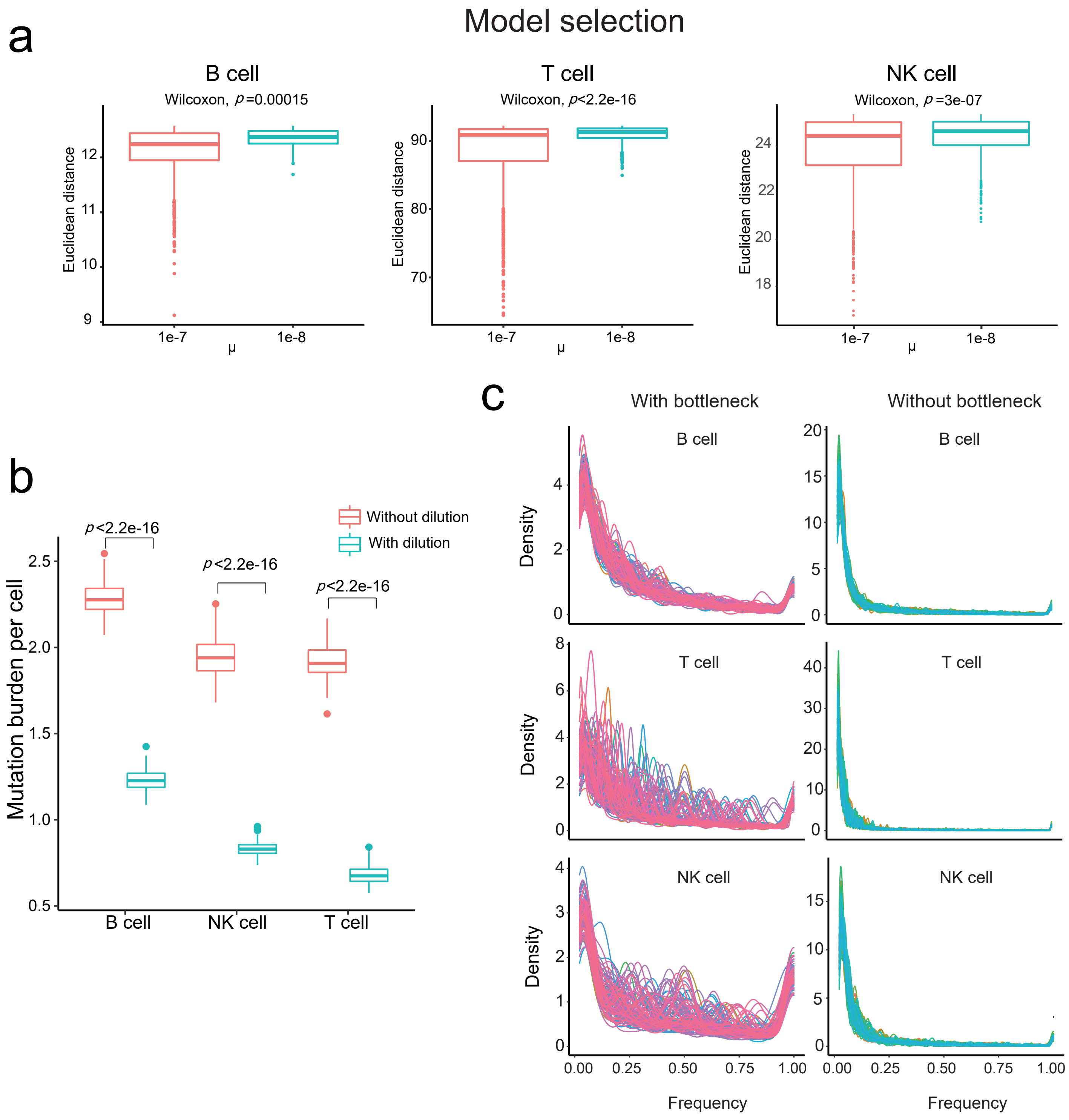

Model selection
a
B cell
Wilcoxon, p=0.00015
T cell
Wilcoxon, p<2.2e-16
NK cell
Wilcoxon, p=3e-07
Euclidean distance
μ
1e-7
1e-8
b
Without dilution
With dilution
p<2.2e-16
p<2.2e-16
p<2.2e-16
Mutation burden per cell
B cell
NK cell
T cell
c
With bottleneck
Without bottleneck
B cell
T cell
NK cell
Density
Frequency

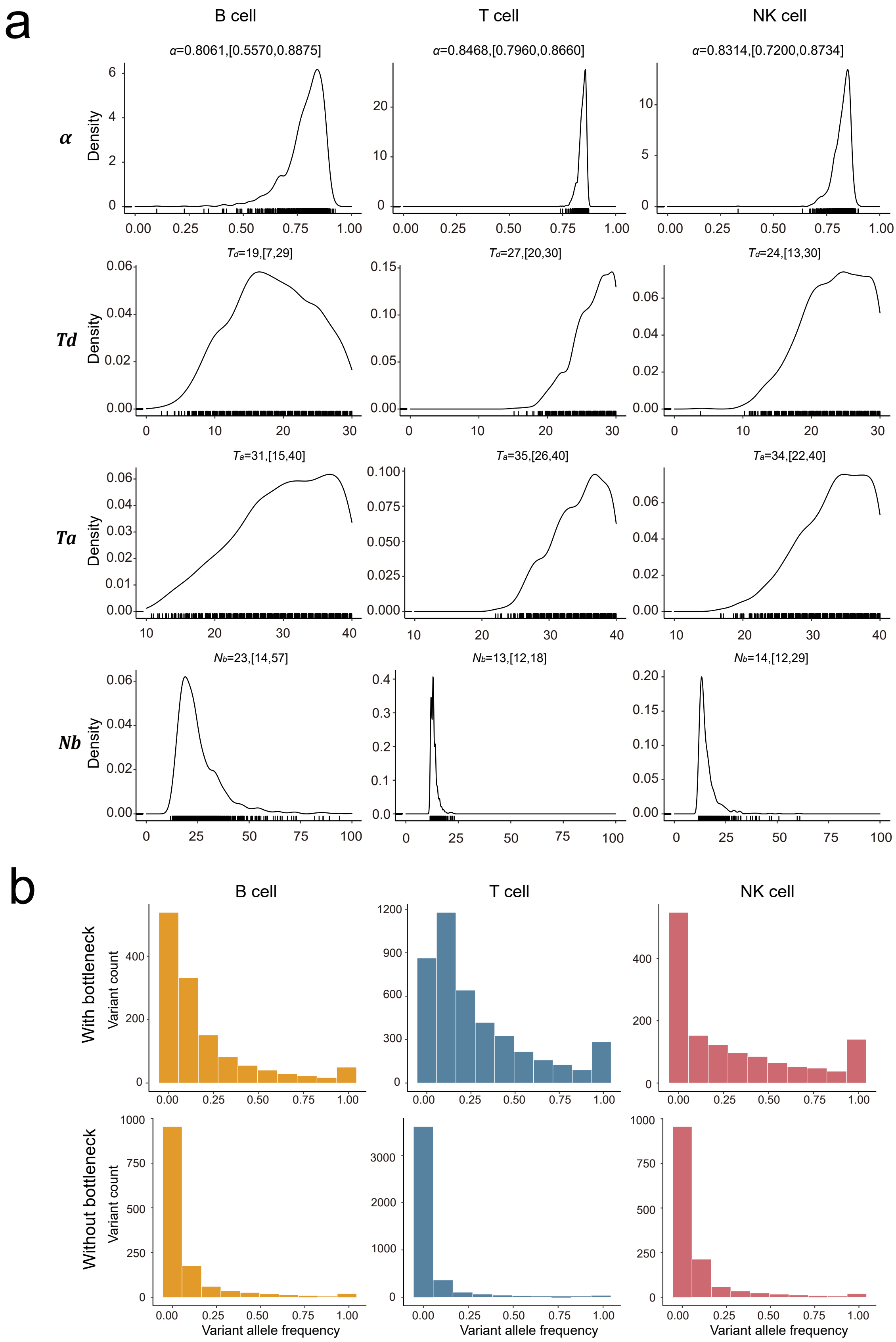
a
B cell
T cell
NK cell
α=0.8061,[0.5570,0.8875]
α=0.8468,[0.7960,0.8660]
α=0.8314,[0.7200,0.8734]
α
Density
Td=19,[7,29]
Td=27,[20,30]
Td=24,[13,30]
Td
Density
Ta=31,[15,40]
Ta=35,[26,40]
Ta=34,[22,40]
Ta
Density
Nb=23,[14,57]
Nb=13,[12,18]
Nb=14,[12,29]
Nb
Density
b
B cell
T cell
NK cell
With bottleneck
Variant count
Without bottleneck
Variant count
Variant allele frequency
Variant allele frequency
Variant allele frequency

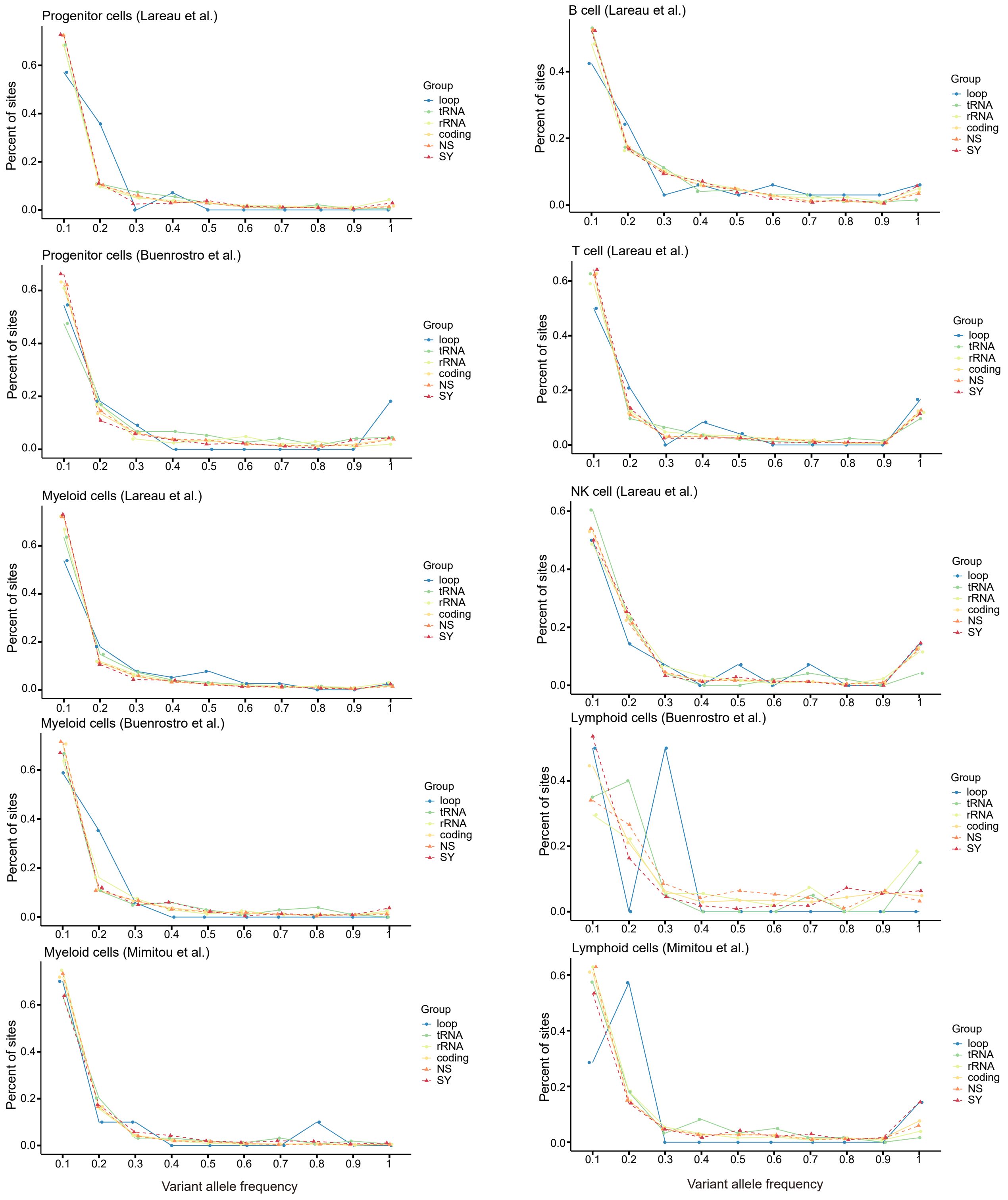
Progenitor cells (Lareau et al.)
B cell (Lareau et al.)
Progenitor cells (Buenrostro et al.)
T cell (Lareau et al.)
Myeloid cells (Lareau et al.)
NK cell (Lareau et al.)
Myeloid cells (Buenrostro et al.)
Lymphoid cells (Buenrostro et al.)
Myeloid cells (Mimitou et al.)
Lymphoid cells (Mimitou et al.)
Percent of sites
Variant allele frequency
Group
loop
tRNA
rRNA
coding
NS
SY
0.0
0.2
0.4
0.6
0.1
0.2
0.3
0.4
0.5
0.6
0.7
0.8
0.9
1

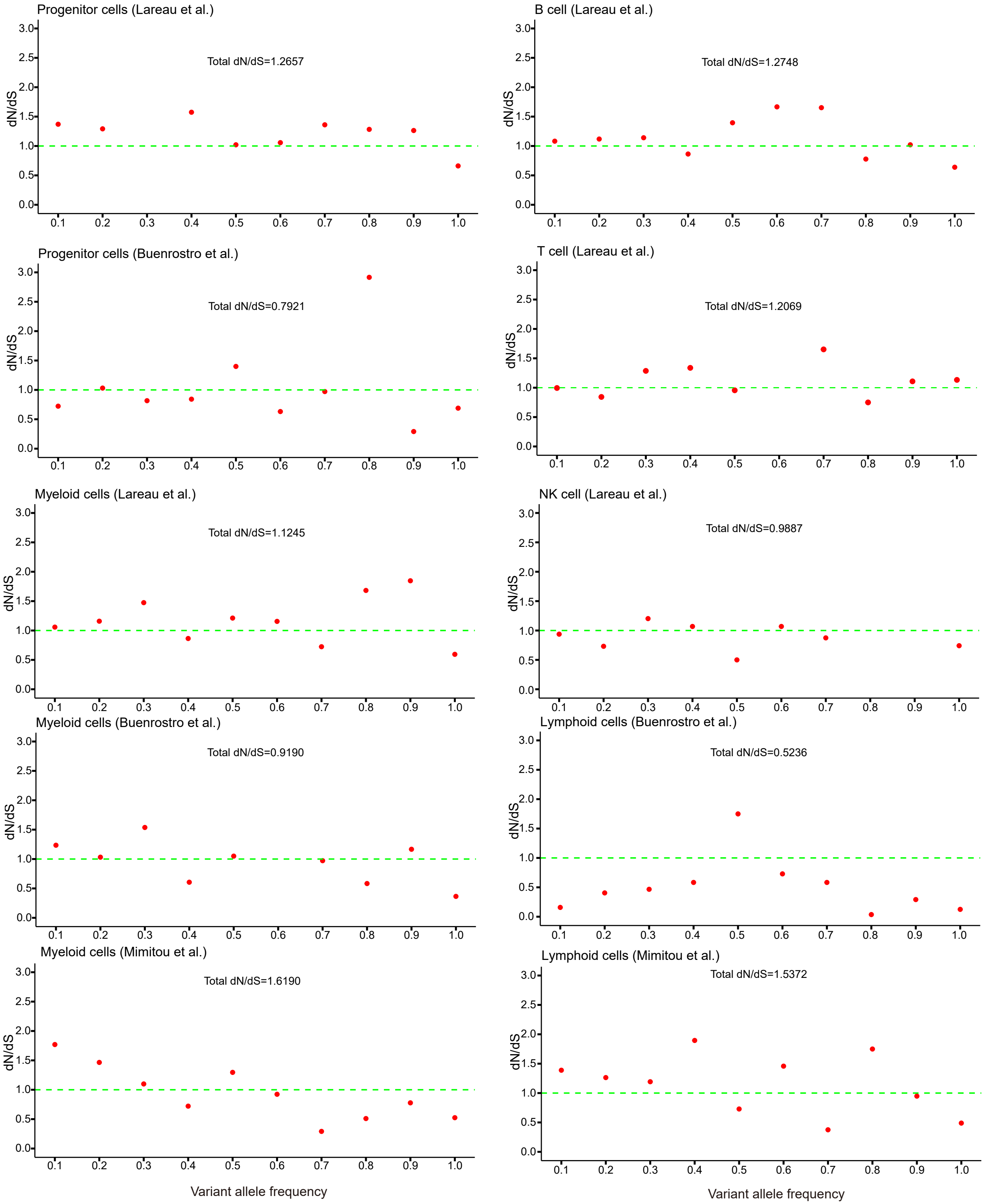

Progenitor cells (Lareau et al.)
Total dN/dS=1.2657
B cell (Lareau et al.)
Total dN/dS=1.2748
Progenitor cells (Buenrostro et al.)
Total dN/dS=0.7921
T cell (Lareau et al.)
Total dN/dS=1.2069
Myeloid cells (Lareau et al.)
Total dN/dS=1.1245
NK cell (Lareau et al.)
Total dN/dS=0.9887
Myeloid cells (Buenrostro et al.)
Total dN/dS=0.9190
Lymphoid cells (Buenrostro et al.)
Total dN/dS=0.5236
Myeloid cells (Mimitou et al.)
Total dN/dS=1.6190
Lymphoid cells (Mimitou et al.)
Total dN/dS=1.5372
dN/dS
0.0
0.5
1.0
1.5
2.0
2.5
3.0
0.1
0.2
0.3
0.4
0.5
0.6
0.7
0.8
0.9
1.0
Variant allele frequency

TableS1-DataResource

Cell Type	DataSet	library	Platform	# of cells	# of individuals	Notes	Author
naïve T cell	GSE107816	scATACseq+ C1	Illumina NextSeq 500	96	1	healthy	Satpathy et al. 2018
naïve T cell (sample 2)	GSE107816	scATACseq+ C1	Illumina NextSeq 500	192	2	healthy	Satpathy et al. 2018
memory T cell	GSE107816	scATACseq+ C1	Illumina NextSeq 500	192	2	healthy	Satpathy et al. 2018
Th17	GSE107816	scATACseq+ C1	Illumina NextSeq 500	192	2	healthy	Satpathy et al. 2018
Multipotent progenitor cell (MPP)	GSE96769	scATACseq+ C1	Illumina NextSeq 500	192	2	BM1077-frozen, BM0828-frozen	Buenrostro et al. 2018
Lymphoid-primed multipotent progenitor (LMPP)	GSE96769	scATACseq+ C1	Illumina NextSeq 500	96	1	BM0828-frozen	Buenrostro et al. 2018
Common lymphoid progenitor (CLP)	GSE96769	scATACseq+ C1	Illumina NextSeq 500	192	2	BM1077-frozen, BM0828-frozen	Buenrostro et al. 2018
Hematopoietic stem cell (HSC)	GSE96769	scATACseq+ C1	Illumina NextSeq 500	480	3	BM1077-Frozen, BM0106-LS/SIM, BM0828-fresh/frozen	Buenrostro et al. 2018
Common myeloid progenitor (CMP)	GSE96769	scATACseq+ C1	Illumina NextSeq 500	672	4	BM1077-frozen/HYC, BM0828-frozen, BM1214-LS, BM1137-LS	Buenrostro et al. 2018
Granulocyte-monocyte progenitors (GMP)	GSE96769	scATACseq+ C1	Illumina NextSeq 500	288	3	BM1077-frozen, BM0828, BM1214	Buenrostro et al. 2018
Plasmacytoid dendritic cell (pDC)	GSE96769	scATACseq+ C1	Illumina NextSeq 500	192	2	BM1137-LS, BM1214-frozen	Buenrostro et al. 2018
CD34+ hematopoietic cells,PBMCs	GSE142745	mtscATAC-seq	NextSeq 550	22312	1	healthy	Lareau et al. 2020
Effector Memory T(Tem)	E-MTAB-6072	Smart-seq2	Illumina HiSeq 2500	64	1	healthy, organism part:colon	Ricardo et al. 2019
Central Memory T(Tcm)	E-MTAB-6072	Smart-seq2	Illumina HiSeq 2500	61	1	healthy, organism part:colon	Ricardo et al. 2019
CD4+ regulatory(Treg)	E-MTAB-6072	Smart-seq2	Illumina HiSeq 2500	131	1	healthy, organism part:colon	Ricardo et al. 2019
PBMCs, BMMCs,CD34+ bone marrow cells	GSE139369	CITE-seq	Illumina NovaSeq 6000	35434	4	healthy	Granja et al. 2019
Bone marrow cells	GSM4732140	mtscATAC-seq	NextSeq 550	10327	1	healthy	Mimitou et al. 2020

TableS2 MarkerGenes(scATAC)	
**Celltype**	**Marker gene for cell type annotation**
HSC/MPP	*AVP,HLF,CRHBP*
LMPP	*FCN2,RUNX1*
CLP	*MME*
proB	*RAG1,CD19,EBF1,IL7R*
preB	*RAG1,CD79B,MS4A1*
Naïve B	*IL4R,PAX5,MS4A1*
Memory B	*PAX5,MS4A1*
plasma	*TACI,BCMA,SDC1,IGKC*
Naive $CD4^+$ T	*ITGA6,CCR7*
Naive $CD8^+$ T	*CD8A,LEF1*
Memory $CD4^+$ T	*CD4,CD52*
Memory $CD8^+$ T	*CD8A,EOMES*
NK	*GNLY,NKG7,FCGR3B,FASLG*
CMP	*TAL1,GATA1*
GMP	*SPI1,MPO*
MDP	*FLT3,MPO*
pDC	*DERL3,FLT3*
Early.ery	*HBB,GATA1*
Late.ery	*GATA1*
Early.baso	*PF4,ITGA2B*
CD14mono	*NCR1,CEBPB*
CD16mono	*FCGR3A,SIGLEC10*

TableS3 MarkerGene(scRNA)	
**Celltype**	**Marker genes for cell type annotation**
HSC/MPP	*AVP,HLF,CRHBP*
LMPP	*FCN2,RUNX1*
CLP	*MME,DNTT,IL7R*
proB	*VPREB3,CD79A,CD79B,RAG1*
preB	*MS4A1,EBF1*
Immature B	*CR2,CD19*
Naïve B	*CD22*
Memory B	*CD27,IL4R*
plasma	*TACI,BCMA,SDC1,IGKC*
Naive $CD4^+$ T	*SELL,CCR7,CD95*
Naive $CD8^+$ T	*LEF1,CD8A,IL2RB*
Memory $CD4^+$ T	*IL7R,CD52,CD4*
Memory $CD8^+$ T	*IFNG,EMOES*
NK	*GNLY,CCL5,CD56,NKG7*
CMP	*TAL1,MPO*
GMP/MDP	*ELANE,MPO,PRTN3,CSF1R*
pDC	*SPIB,DERL3,IRF8*
cDC	*CDC1,SPI1*
Early.ery	*HBB,GATA1*
Late.ery	*HBB,BLVRB*
Early.baso	*BCVRB , ITGA2B,PF4,LMO4*
CD14mono	*CD14*
CD16mono	*FCGR3A,SIGLEC10*
