# Supplementary material for: A Genetic Bottleneck of Mitochondrial DNA During Human Lymphocyte Development
Source: Mol Biol Evol. 2022 Apr 28;39(5):msac090. doi: 10.1093/molbev/msac090 (PMC9113143; doi:10.1093/molbev/msac090)
Supplement: msac090_Supplementary_Data [file msac090_supplementary_data.zip › Supplementary_Figures_and_Legends.docx]

**
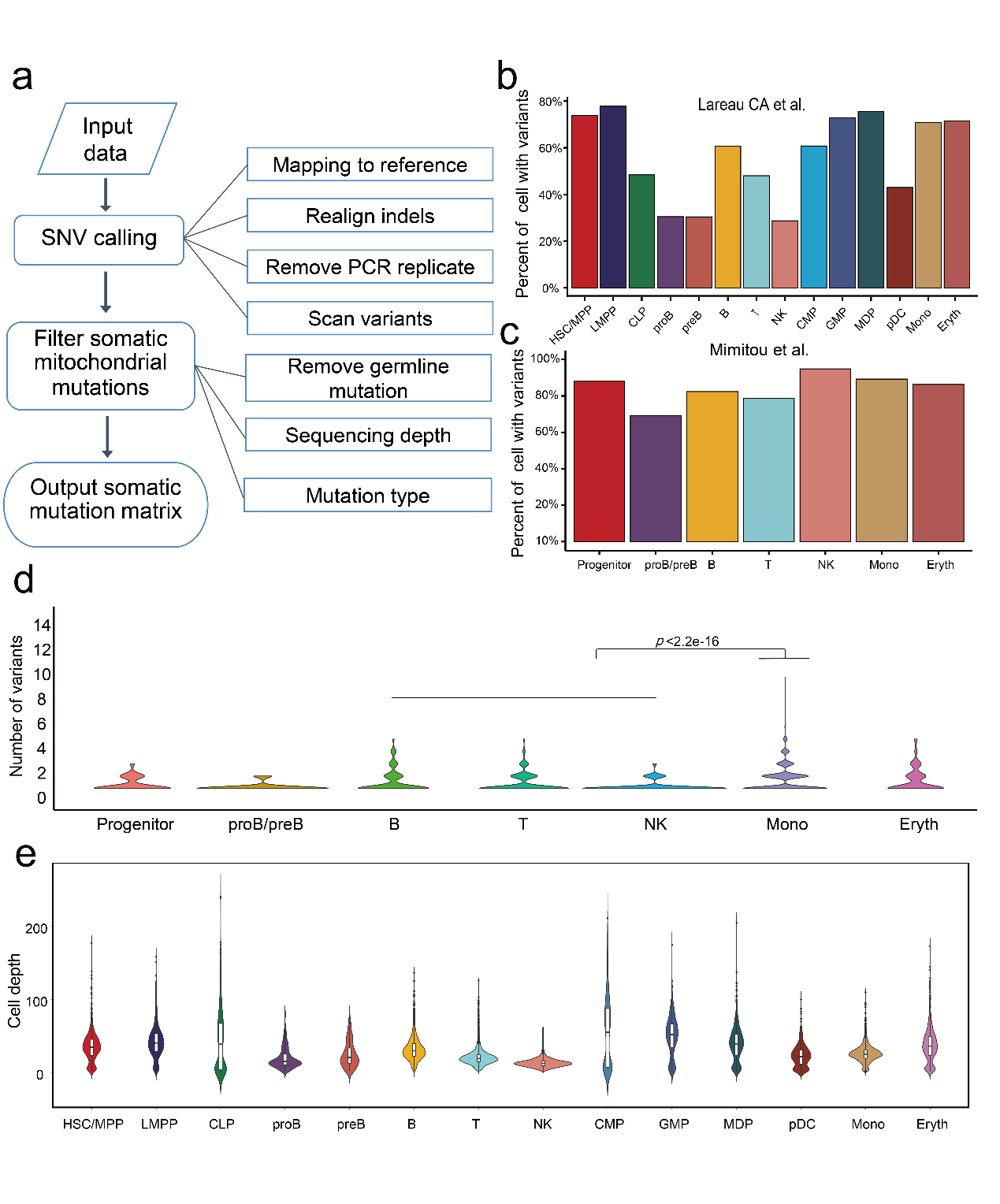
**

**Supplementary Fig. 1 Detection of somatic mitochondrial mutations in single PBMCs with scATAC-seq data or scRNA-seq data.**

(**a**) Schematic of mtDNA mutation calling with scATAC-seq (including mtscATAC-seq) or scRNA-seq data.

(**b**) Percentage of cells with at least one somatic mtDNA mutation detected in individual cells for each cell type in the mtscATAC-seq data from Lareau et al.

(**c**) Percentage of cells with at least one somatic mtDNA mutation detected in individual cells for each cell type in the mtscATAC-seq data from Mimitou et al.

**(d)** Violin plot showing the number of somatic mtDNA variants per cell for various cell types; *p* values based on a two-sided Wilcoxon rank-sum test are as shown.

**(e)** Violin plot showing the average depth per cell for each cell type.

**
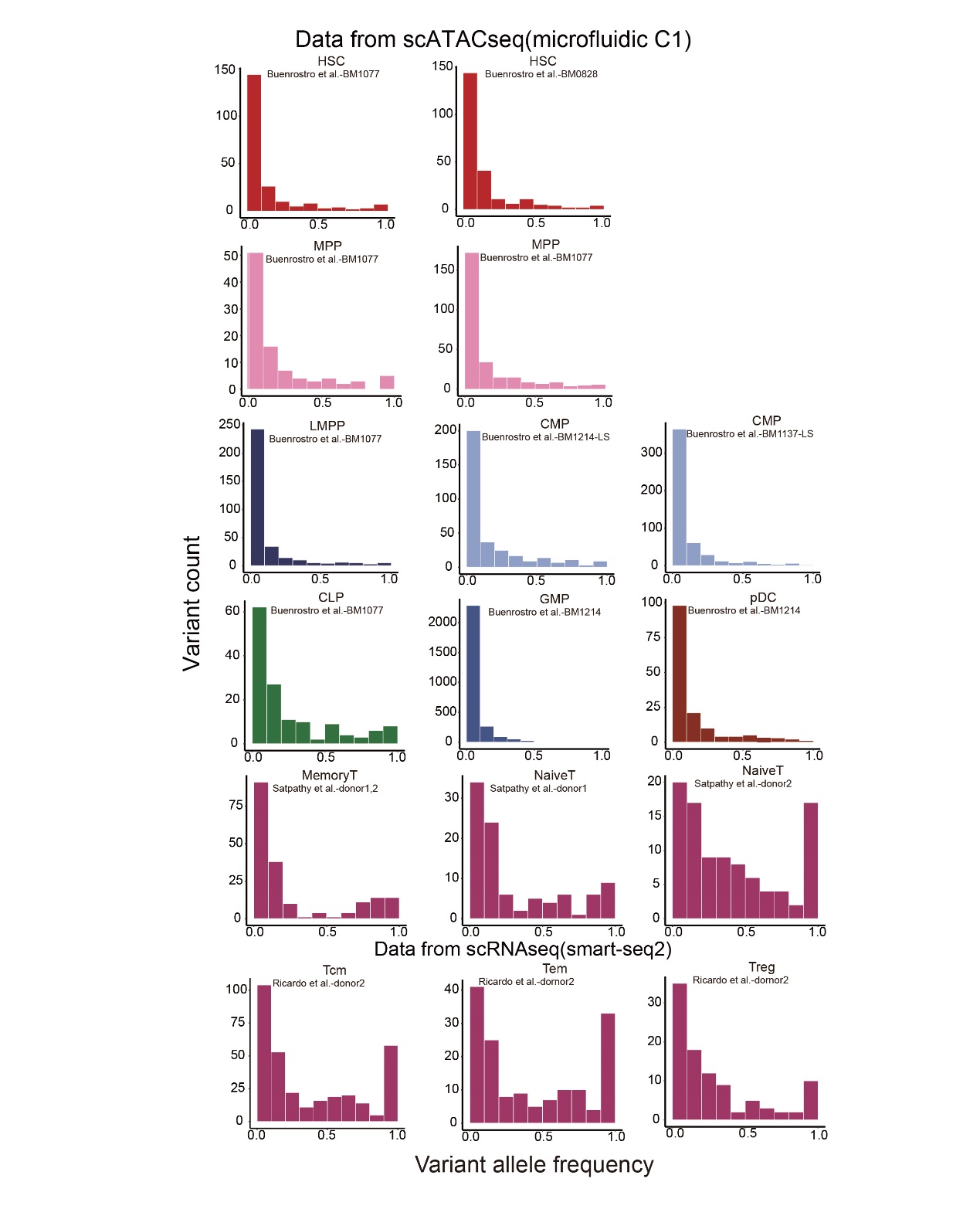
**

**Supplementary Fig. 2**

Allele frequency spectrum of somatic mtDNA mutations for different hematopoietic cell types, based on independent scATAC datasets (Buenrostro et al. and Satpathy et al.) and an scRNA-seq dataset (Ricardo et al.).

**
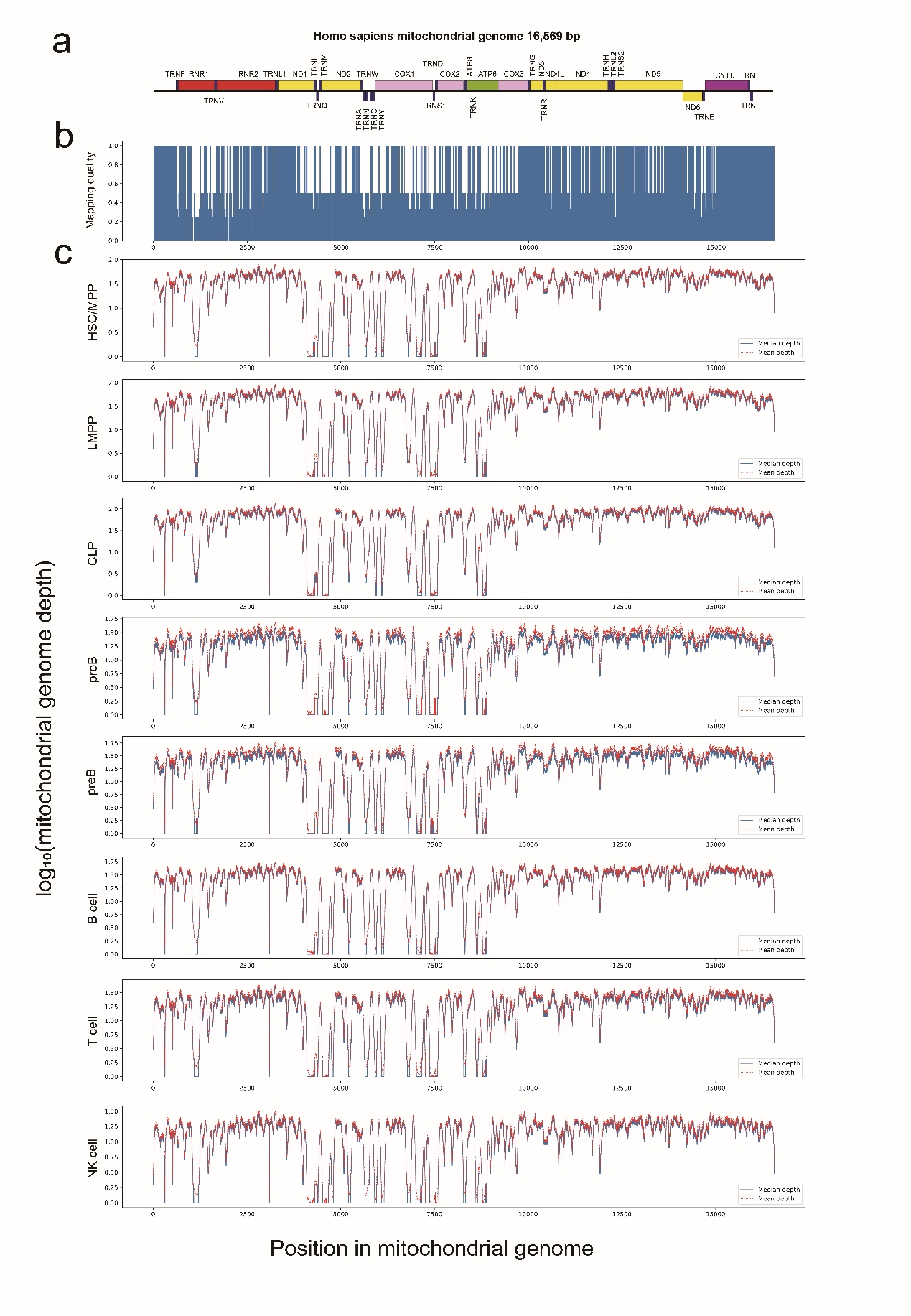
**

**Supplementary Fig. 3 Sequencing depth by site along the mitochondrial genome**

**(a)**The human mitochondrial DNA genome consists of 16569 bp encoding 13 proteins, 2 ribosomal RNAs and 22 transfer RNAS (on the top).

**(b)**The x-axis shows the nucleotide position on the mitochondrial genome. The mapping quality score (generated by ENCODE project) represents the probability of uniquely align to a region with 35-mer sequences.

**(c)**The x-axis shows the nucleotide position on the mitochondrial genome and the y-axis represents the median (blue line) and mean (red line) sequencing depth across cells in each cell type along lymphoid lineage.

**
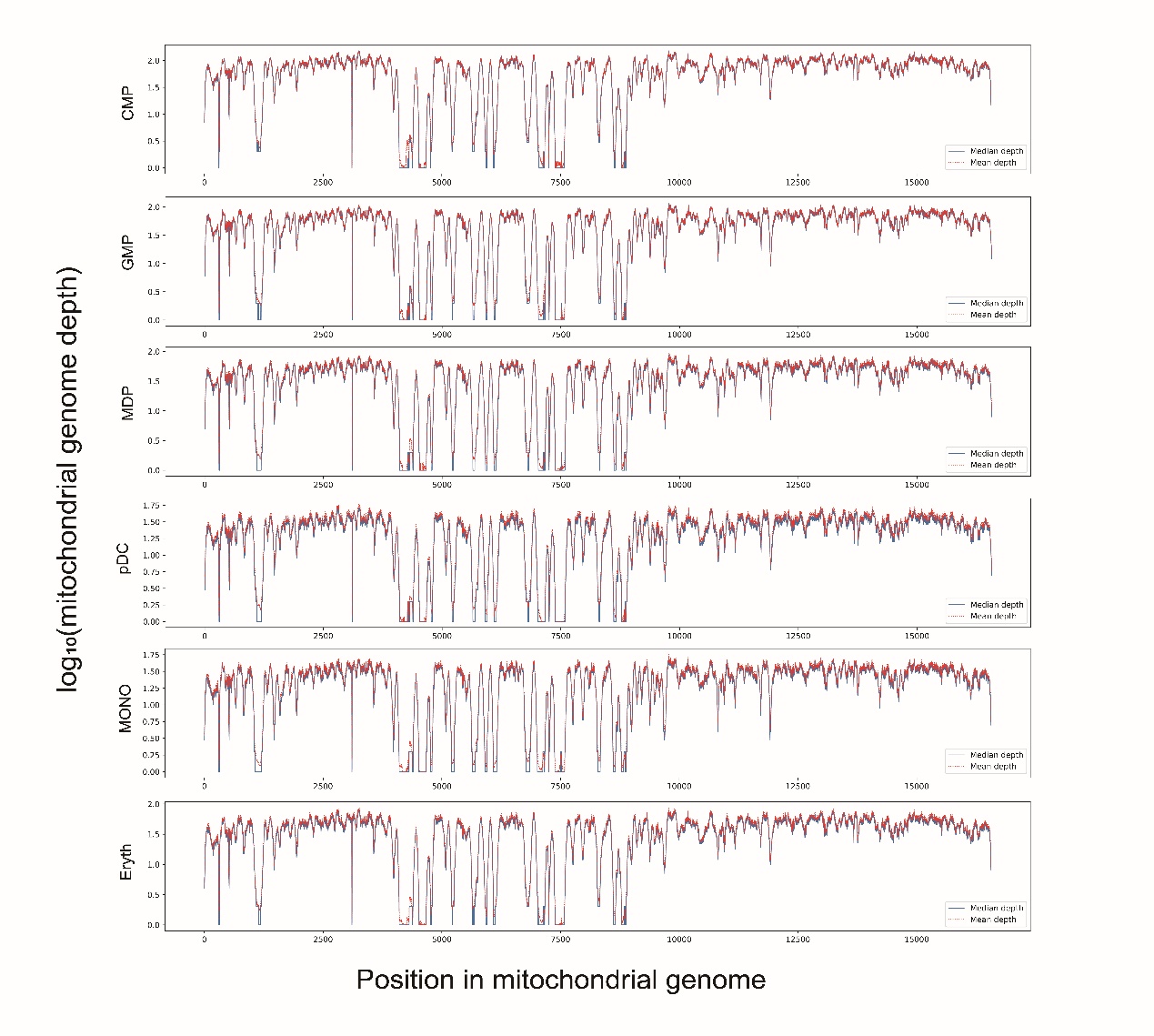
**

**Supplementary Fig. 4 Sequencing depth by site along the mitochondrial genome.**

The x-axis shows the nucleotide position on the mitochondrial genome and the y-axis represents the median (blue line) and mean (red line) sequencing depth across cell in each cell type along myeloid lineage.

**
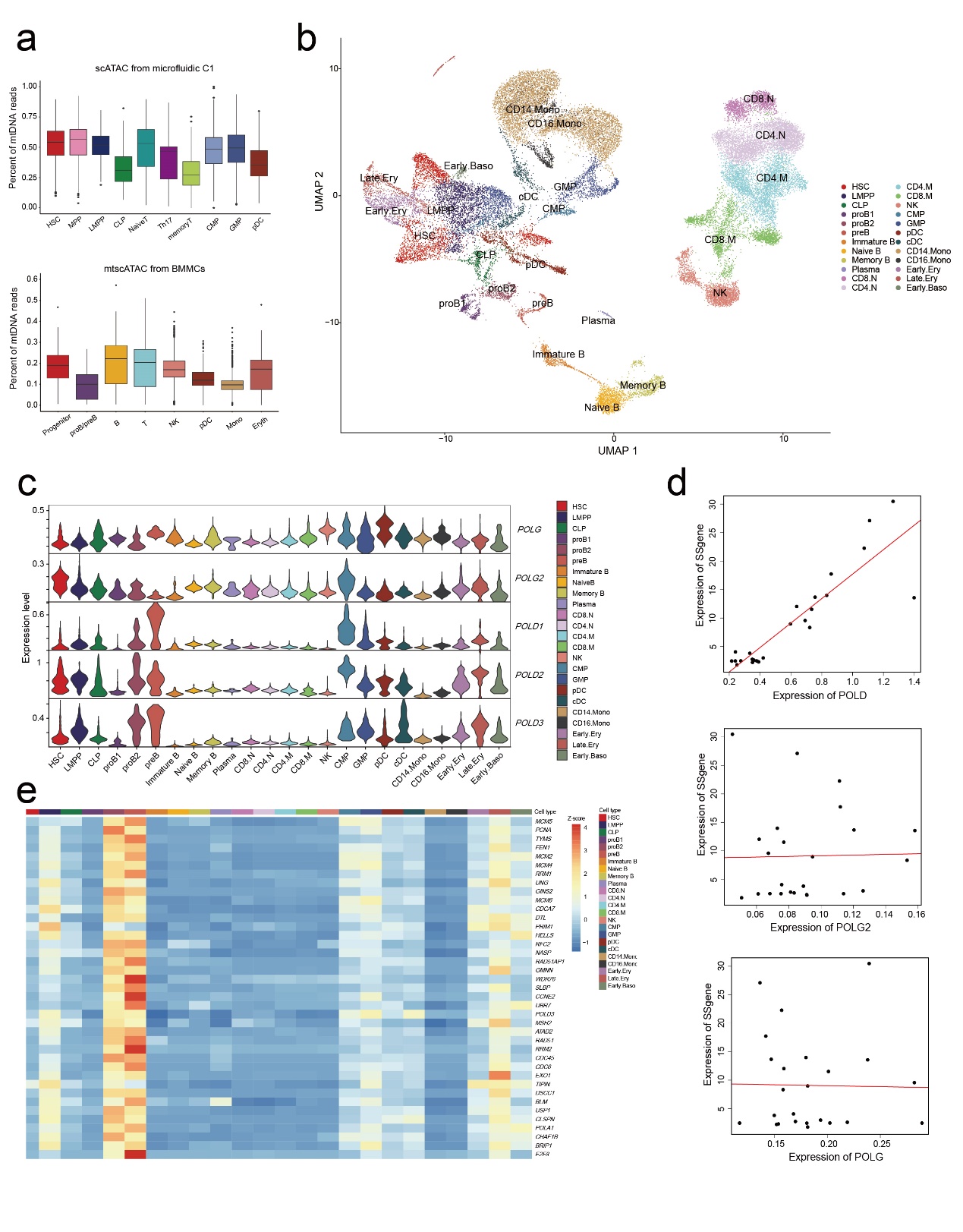
**

**Supplementary Fig. 5 Gene expression of G1/S phase-specific genes in scRNA-seq data.**

(**a**) Relative mtDNA copies were measured by the percentage of the sequencing reads mapped to the mitochondrial genome out of the total number of reads for each cell types.

(**b**) UMAP projection of PBMCs, BBMCs and CD34^+^ PBMCs with scRNA-seq data. Dots represent individual cells colored by cell types.

(**c**) Violin plots showing the expression of mitochondrial DNA polymerase γ (*POLG*) and its binding subunit (*POLG2*) and nuclear DNA replication polymerase genes (*POLD1–3*) from scRNA-seq data.

(**d**) Scatter plot showing the correlation of the gene expression of *POLD* (*POLD1–3*), *POLG* and *POLG2* with G1/S phase-specific genes (SSgene).

(**e**) Heat map showing the expression of 39 G1/S phase-specific genes in 24 cell types.

**
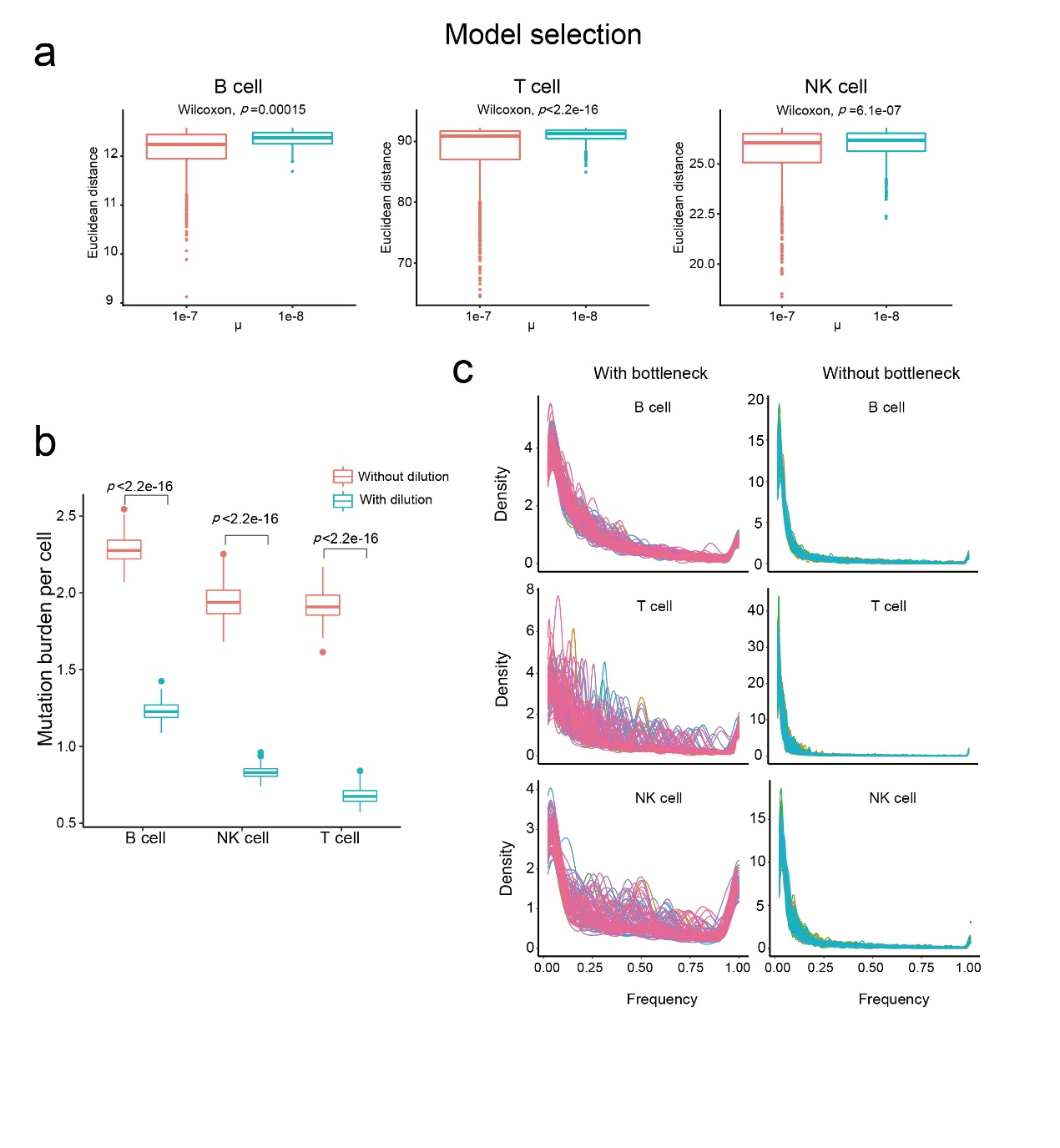
**

**Supplementary Fig. 6 Details regarding the parameter inference for the dilution model of the mitochondrial genetic bottleneck.**

**(a)** Model selection with respect to the per-site mutation rate 𝜇. We ran the ABC inference procedures for two mutation rates $\mu$=10^-8^ and 10^-7^, and $\mu$=10^-7^ fitted the data better (smaller Euclidean distance between simulated and observed summary statistics) in all cell types and thus was used for the parameter inference.

(**b**) Simulations under the dilution model of the mitochondrial genetic bottleneck with the ABC-estimated parameter values recapitulated the lower mutation burden in B, T and NK cells, as compared with simulations without a mitochondrial genetic bottleneck.

(**c**) Simulations (100 times) with inferred parameters from the dilution model under conditions with or without a mitochondrial genetic bottleneck. Each curve represents one simulation.

**
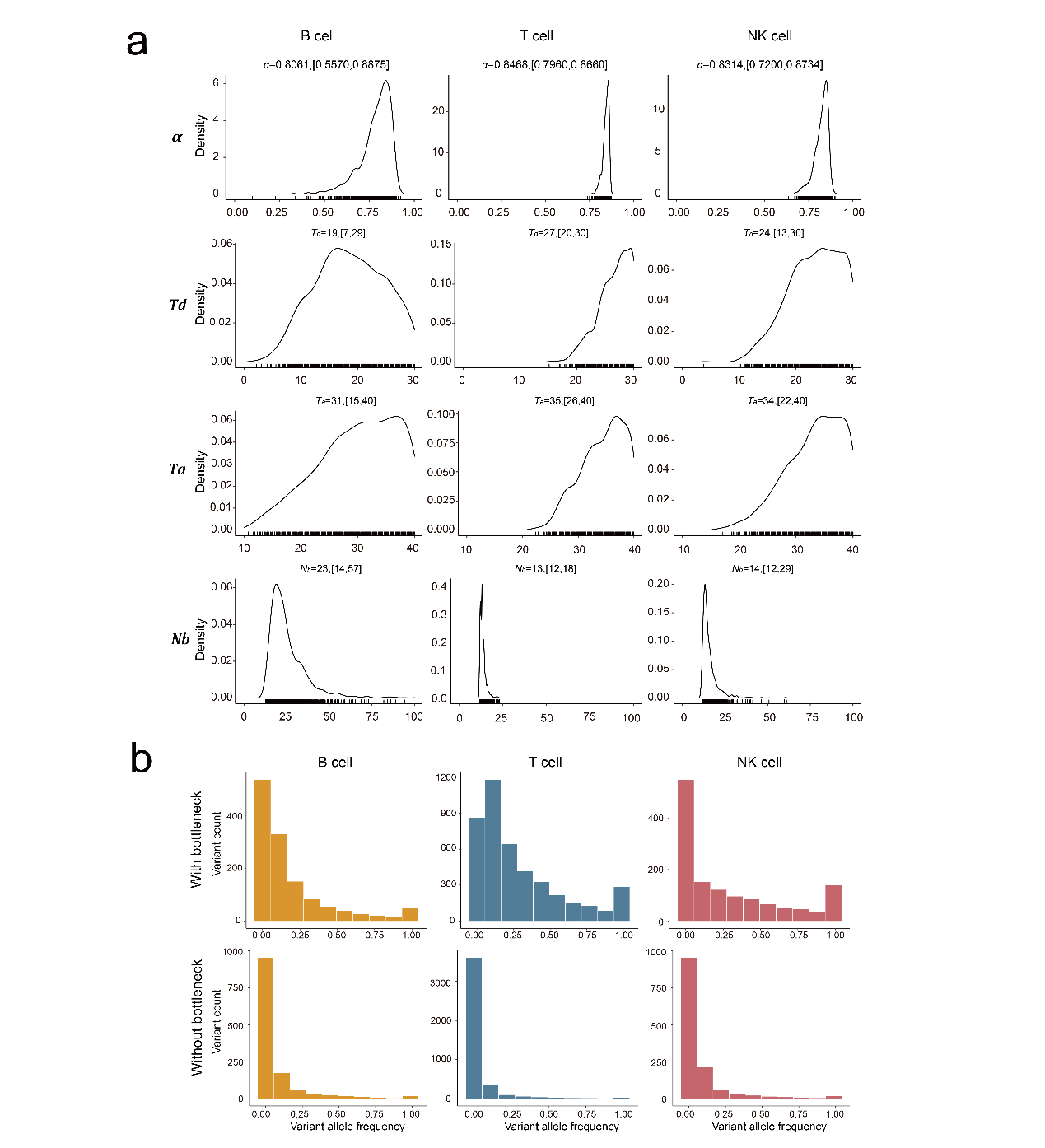
**

**Supplementary Fig. 7 The dilution model of the mitochondrial genetic bottleneck with start 1000 mtDNA copy number.**

**(a)** The distribution of model parameters inferred by the Approximate Bayesian Computation (ABC) algorithm. The starting mtDNA copies of the model is 1000. The mean and 95% confidence interval of each parameter estimation is as shown.

**(b)** Simulations based on the dilution model of mitochondrial genetic bottleneck with the ABC-estimated parameter values recapitulated the lymphocyte-specific overrepresentation of homoplasmic mutations. The upper and lower panels represent the simulations with and without mitochondrial genetic bottleneck, respectively.

**
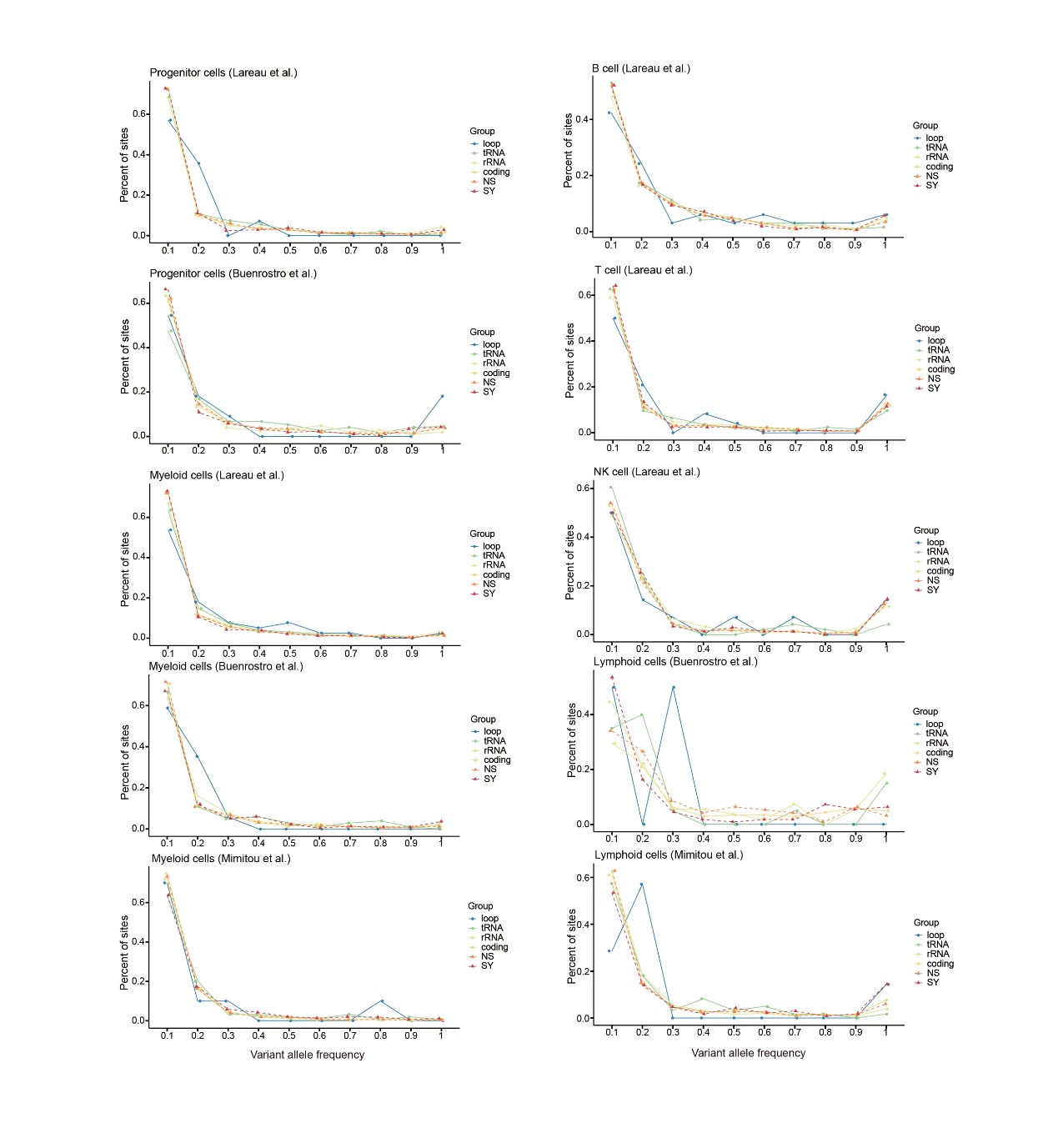
**

**Supplementary Fig. 8 Allele frequency spectrum of somatic mtDNA mutations for different types in the hematopoietic system.**

Distribution of the VAF for mutations in different mtDNA genomic regions or types in progenitor and myeloid cells. The color code corresponds to mtDNA genomic regions or mutation types, annotated as loop, tRNA, rRNA, coding (coding region), NS (non-synonymous) and SY (synonymous).

**
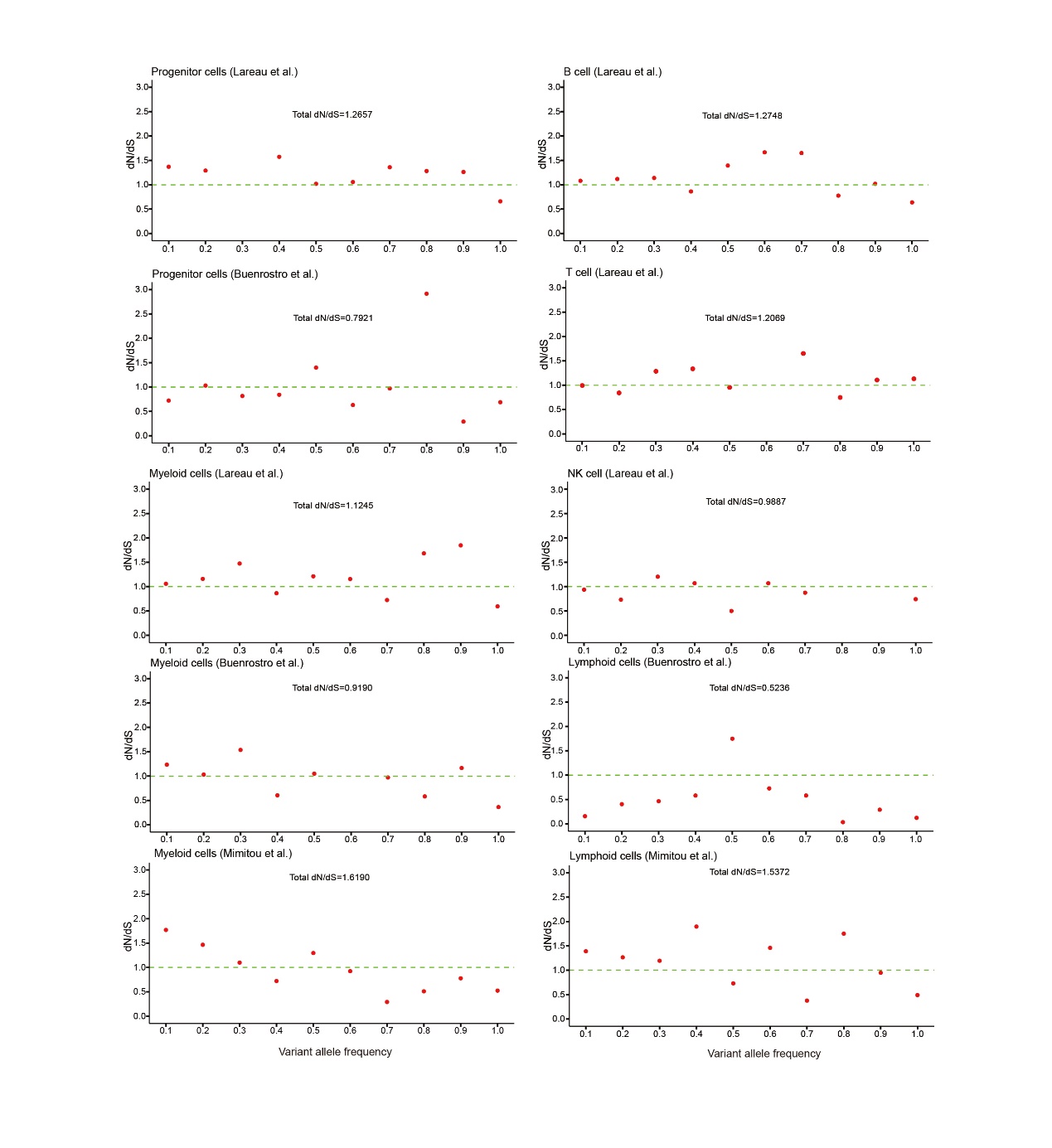
**

**Supplementary Fig. 9 dN/dS ratio for different types.**

dN/dS ratio (y-axis) for mutations in different VAF bins (x-axis).

**Supplementary Table 1**

**
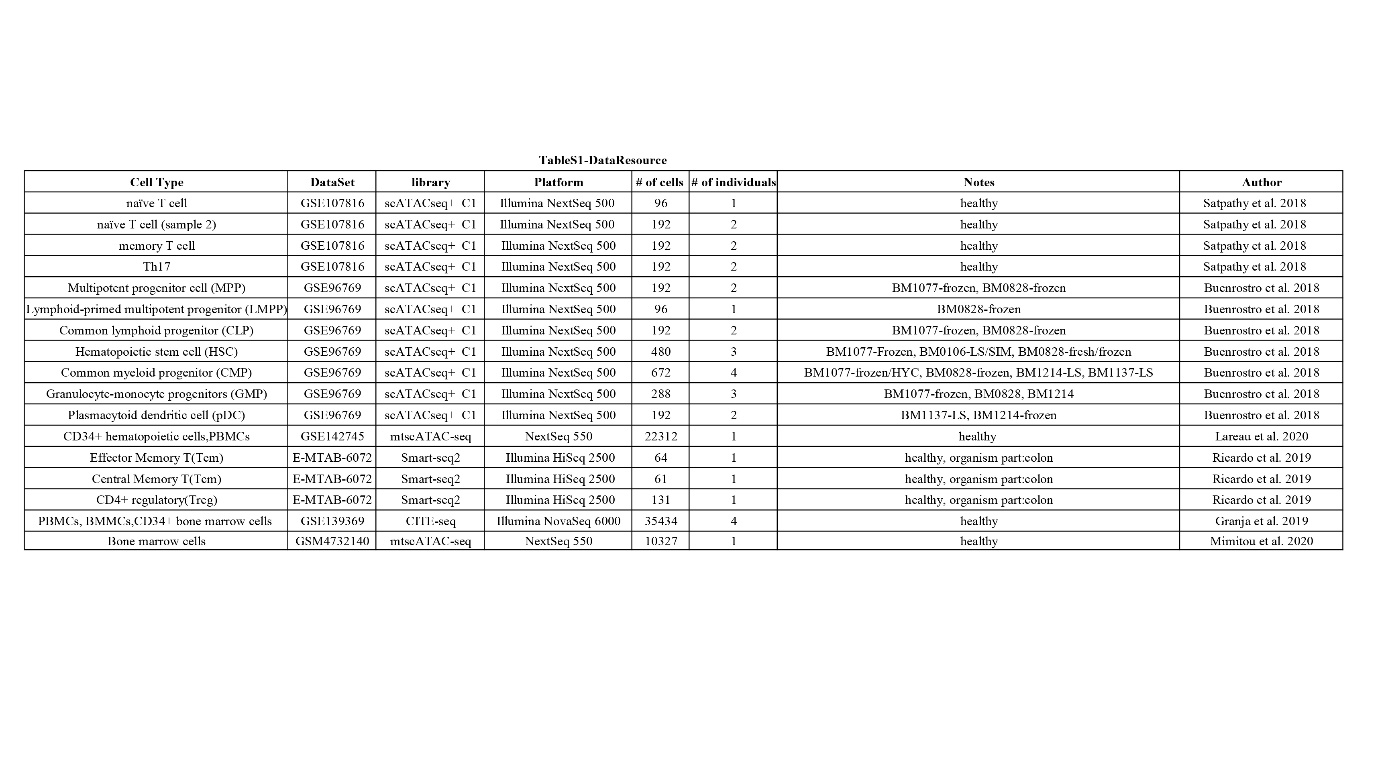
**

**Supplementary Table 2**

**
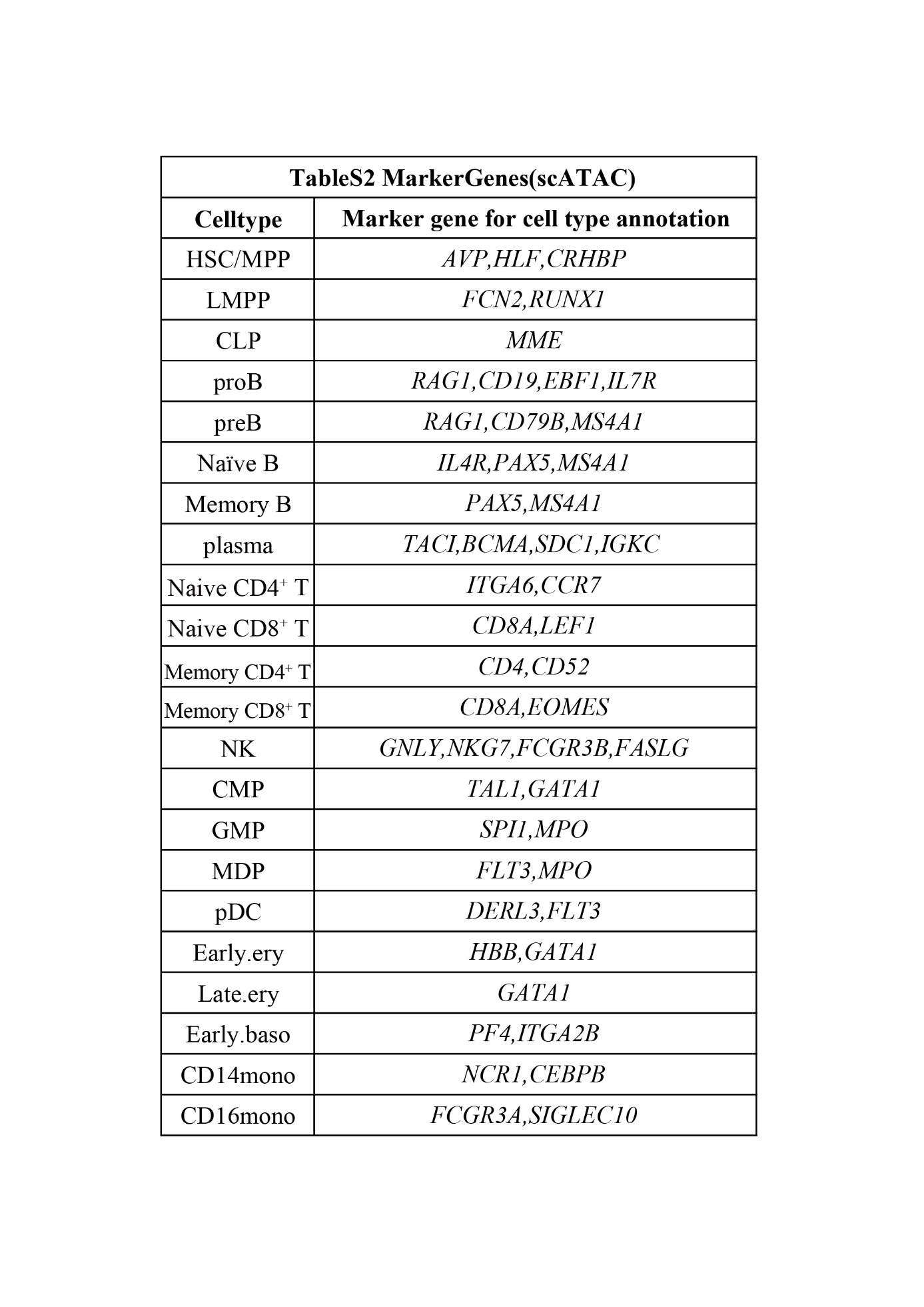
**

**Supplementary Table 3**

**
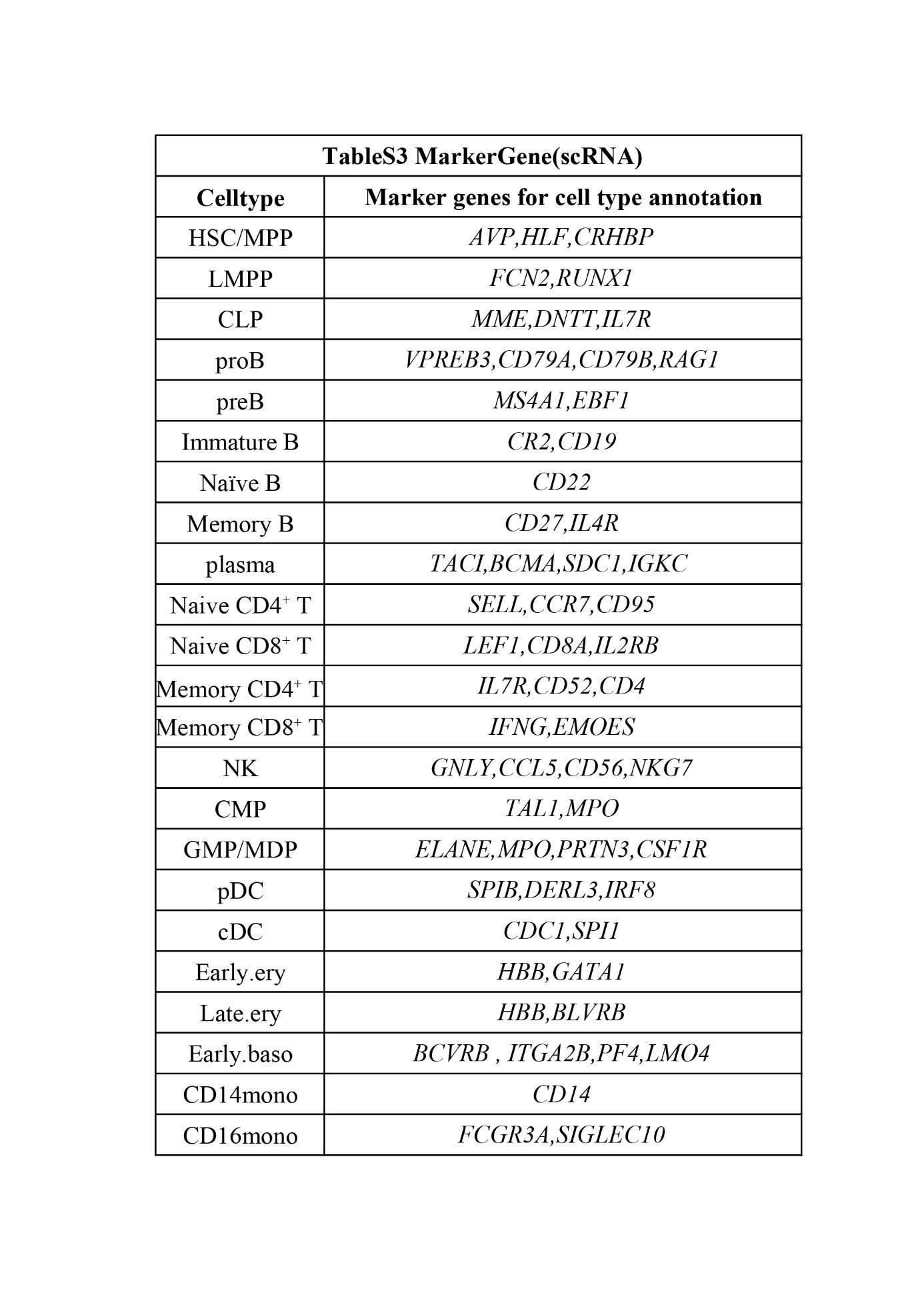
**
